# Sequencing and Analysis of *Strobilanthes cusia* (Nees) Kuntze Chloroplast Genome Revealed the Rare Simultaneous Contraction and Expansion of the Inverted Repeat Region in Angiosperm

**DOI:** 10.3389/fpls.2018.00324

**Published:** 2018-03-14

**Authors:** Haimei Chen, Junjie Shao, Hui Zhang, Mei Jiang, Linfang Huang, Zhao Zhang, Dan Yang, Molly He, Mostafa Ronaghi, Xi Luo, Botao Sun, Wuwei Wu, Chang Liu

**Affiliations:** ^1^Key Laboratory of Bioactive Substances and Resource Utilization of Chinese Herbal Medicine, Ministry of Education, Institute of Medicinal Plant Development, Chinese Academy of Medical Sciences and Peking Union Medical College, Beijing, China; ^2^Illumina, Inc., San Diego, CA, United States; ^3^Guangxi Botanical Garden of Medicinal Plants, Nanning, China

**Keywords:** Ban-Lan, Ban-Lan-Gen, *Strobilanthes cusia* (Nees) Kuntze, *Baphicacanthus cusia* (Nees) Bremek, chloroplast genome

## Abstract

Ban-Lan-Gen, the root tissues derived from several morphologically indistinguishable plant species, have been used widely in traditional Chinese medicines for numerous years. The identification of reliable markers to distinguish various source plant species is critical for the effective and safe use of products containing Ban-Lan-Gen. Here, we analyzed and characterized the complete chloroplast (cp) genome sequence of *Strobilanthes cusia* (Nees) Kuntze to identify high-resolution markers for the species determination of Southern Ban-Lan-Gen. Total DNA was extracted and subjected to next-generation sequencing. The cp genome was then assembled, and the gaps were filled using PCR amplification and Sanger sequencing. Genome annotation was conducted using CpGAVAS web server. The genome was 144,133 bp in length, presenting a typical quadripartite structure of large (LSC; 91,666 bp) and small (SSC; 17,328 bp) single-copy regions separated by a pair of inverted repeats (IRs; 17,811 bp). The genome encodes 113 unique genes, including 79 protein-coding, 30 transfer RNA, and 4 ribosomal RNA genes. A total of 20 tandem, 2 forward, and 6 palindromic repeats were detected in the genome. A phylogenetic analysis based on 65 protein-coding genes showed that *S. cusia* was closely related to *Andrographis paniculata* and *Ruellia breedlovei*, which belong to the same family, Acanthaceae. One interesting feature is that the IR regions apparently undergo simultaneous contraction and expansion, resulting in the presence of single copies of rps19, rpl2, rpl23, and ycf2 in the LSC region and the duplication of psbA and trnH genes in the IRs. This study provides the first complete cp genome in the genus *Strobilanthes*, containing critical information for the classification of various *Strobilanthes* species in the future. This study also provides the foundation for precisely determining the plant sources of Ban-Lan-Gen.

## Introduction

Ban-Lan-Gen, the root tissues of several morphologically indistinguishable plant species known as Ban-Lan, is used in various traditional Chinese medicine (TCM) products for the treatment of viral hepatitis, influenza, cold, pneumonia, inflammation, herpes, erysipelas, and snakebite (Chinese Pharmacopoeia Commission, 2015). Chemical components with diverse structures, including indole alkaloids, quinazolinone alkaloids, monoterpenes, triterpenes, flavonoids, sterols, anthraquinones, benzoxazinones, and lignans, are derived from the extracts of Ban-Lan-Gen (Honda and Tabata, 1979; Sun et al., 2008; Gu et al., 2014). Pharmacological studies indicate that these components exert antimicrobial, antiviral, anticancer (Merz et al., 2004; Ichimaru et al., 2015), and anti-inflammatory effects (Otsuka et al., 1988).

Similar to those of numerous TCMs, the exact identities of the source plants of Ban-Lan-Gen are usually not well defined. Since 1995, Pharmacopoeia of the People’s Republic of China has defined *Isatis indigotica* Fort as the source plant of Northern Ban-Lan-Gen and *Baphicacanthus cusia* (Nees) Bremek as the source plant of Southern Ban-Lan-Gen. However, people still use other plants as source of Ban-Lan-Gen. For example, according to Flora of China^1^, species including *Strobilanthes cusia* (Nees) Kuntze, *Strobilanthes balansae* Lindau, *Ruellia indigotica* Fortune, *Ruellia indigofera* Griff*., Goldfussia cusia* Nees, *Dipteracanthus calycinus* Champion ex Bentham, *Strobilanthes championii* T. Anderson, and *Strobilanthes flaccidifolia* Nees are all considered the aliases of *B. cusia* (Nees) Bremek. Another study stated that “Ban-Lan-Gen is the common name for the dried roots of indigo plants, including *Polygonum tinctorium*, *Isatis indigotica*, *Isatis tinctoria*, and *Strobilanthes cusia*.” (Kumagai et al., 2016) The confusion regarding source plants with the common name “Ban-Lan” has undermined the consistent chemical compositions for medicinal products that use Ban-Lan as its source materials, leading to serious problems in the efficacy and safety of the medicinal products. Therefore, the identification of molecular markers that can unambiguously distinguish the plant materials is critical for ensuring the beneficial effects of medicinal products, including “Ban-Lan.”

*S. cusia* (Nees) Kuntze, also known as *B. cusia* (Nees) Bremek and commonly called Ban-Lan, is a member of the family Acanthaceae. This herbaceous plant is considered as the typical species for Southern Ban-Lan-Gen (Chinese Pharmacopoeia) and native to northeast India, Myanmar, Thailand, and Southern China. The plant has been widely used as a dye and the source of traditional herbal medicines (Wu et al., 2006). Whereas its roots are commonly known as Ban-Lan-Gen and used as medicinal materials, the stems and leaves of *S. cusia* are used as the dye known as indigo blue (Liu et al., 2014). However, the species identity of *S. cusia* is not well defined. The genus *Strobilanthes* contains approximately 400 species; their morphological characters are highly homoplasious, causing difficulty in further classification and differentiation of various *Strobilanthes* species. The lacks of suitable samples and sufficient sampling have also hindered the usage of molecular markers for the phylogenetic classification of *Strobilanthes* species.

The chloroplast (cp) is an organelle that sustains life on earth by converting solar energy to carbohydrates through photosynthesis and oxygen release. The cp genome is ideal for ecological, evolutionary, and diversity studies due to several biological properties, such as high copy numbers of the genome compared with the nuclear genome, conserved genome structure, uniparental inheritance, and low effective population sizes (Wicke et al., 2011). Thus, the sequencing of plastid locus is instrumental in improving our understanding of phylogenetic relationships (Jansen et al., 2007), phylogeographic patterns (Golden and Bain, 2000), species discrimination (Nock et al., 2011), hybridization (Palme et al., 2004), photosynthesis (Leister, 2003), and genome evolution (Wicke et al., 2011, 2016) for particular taxonomic groups. Although single-gene regions are the workhorse for molecular phylogenetic studies, the move from analysis of single-gene regions, which can be amplified by polymerase chain reaction (PCR), to complete cp genome is important to provide subspecies or variety-level resolution and address previously unanswered questions. The presence of hyper-variable sites suggests that sequences derived from cp genome can potentially be used to discriminate closely related species (Lei et al., 2016). As a result, obtaining the cp genome sequences of plant materials used for Ban-Lan-Gen can likely determine the exact species for the materials.

At present, more than one thousand cp genome sequences have been deposited at the National Center for Biotechnology Information (NCBI), including all of the major lineages of the plant kingdom. However, only two complete cp genomes of Acanthaceae, namely *Andrographis paniculata* and *Ruellia breedlovei*, have been sequenced. Moreover, no cp genome is available for species from the *Strobilanthes* genus. In this study, we sequenced and assembled the complete cp genome of *S. cusia* to identify molecular markers that can be used to classify *S. cusia* and its closely related species and to understand their phylogenetic relationships. The genome was then subjected to detailed analysis to identify genes and repeat elements. One interesting feature of the cp genome is that the inverted repeat (IR) regions showed simultaneous contraction and expansion, which is a rather rare observation in angiosperms. In summary, results obtained in this study provided valuable information to elucidate the evolutionary history of species in Acanthaceae. In addition, these results laid the foundation for the precise determination of plant species for the materials used in Ban-Lan-Gen.

## Materials and Methods

### Plant Material and DNA Purification

Fresh leaves of *S. cusia* from multiple individuals were collected from Guangxi Medicinal Plant Garden, Nanning, Guangxi, China and stored at 4°C for cp DNA isolation. *S. cusia* is not an endangered or protected species, and no specific permissions were required for collection. A voucher specimen was deposited at the Institute of Medicinal Plant Development, Beijing, China. Total DNA was extracted using a plant genomic DNA kit (Tiangen Biotech, Beijing, Co., Ltd.). DNA purity was evaluated using 1.0% agarose gel, and DNA concentration was measured using a Nanodrop spectrophotometer 2000 (Thermo Fisher Scientific, United States).

### Genome Sequencing, Assembly, and Annotation

Further steps of library preparation were performed using a TruSeq DNA Sample Prep Kit (Illumina, Inc., United States) according to the manufacturer’s instructions. The DNA was sheared to yield approximately 500 bp-long fragments for paired-end library construction. The library was sequenced on Illumina HiSeq 3000 (Illumina Inc.). In total, 9,912,889 paired-end reads (2 × 150 bp) were obtained.

We first downloaded 1,006 plastid genomes from GenBank in February 2016. These plastid genome sequences were used to search against Illumina paired-end reads using BLASTN with an E-value cutoff of 1e-5. The genome sequence of *A. paniculata* (Accession number: NC_022451) had the highest overall sequence similarity to the reads and was used as a reference for the downstream genome assembly.

AbySS (v1.5.2) (Simpson et al., 2009) and CLC Genomics Workbench (v7) was used for the *de novo* genome assembly. Using Gepard (Krumsiek et al., 2007), we identified 7 contigs from the assembly of contigs of AbySS and CLC Genomics Workbench, respectively, that nearly spanned the entire cp genome. All the 14 contigs were assembled using Seqman module of DNASTAR (v11.0). Then, we obtained only one sequence corresponding to the large single-copy (LSC), IR, and small single-copy (SSC) regions of *S. cusia.* Regions corresponding to the IR/SSC and IR/LSC boundaries were confirmed via direct PCR amplifications.

PCR amplifications were performed using the sequence-specific primers (Supplementary Table S1) under the following conditions: pre-denaturation at 94°C for 2 min, 35 cycles of amplification at 94°C for 30 s, 55°C for 30 s, and 72°C for 30 s, followed by a final extension at 72°C for 2 min. The PCR reaction mixture contained 25 μL of Taq MasterMix (2 × ), 2 μL of forward primer (10 μM), 2 μL of reverse primer (10 μM), and purified cp DNA ( < 1 μg). RNase-free water was added to a final reaction volume of 50 μL.

The CpGAVAS web service (Liu et al., 2012) was used to annotate the *S. cusia* cp genome. Cutoffs for the E-values of BLASTN and BLASTX were 1e-10. The number of top hits to be included in the reference gene sets for annotation after the pre-filtering step was 10. Meanwhile, tRNA genes were identified using tRNAscan-SE (Lowe and Eddy, 1997) and ARAGORN (Laslett and Canback, 2004). Manual corrections on the positions of the start and stop codons, and for the intron/exon boundaries were performed based on the entries in the cp genome database (Cui et al., 2006) using the Apollo program (Lee et al., 2009). Moreover, the circular cp genome map of *S. cusia* was drawn using OrganellarGenomeDRAW (Lohse et al., 2013). Furthermore, codon usage and GC content (that is, the percentage of Guanines and Cytosines) were analyzed using the Cusp and Compseq programs provided by EMBOSS (Rice et al., 2000). Final genome assembly and genome annotation results were deposited in the GenBank (accession number: MG874806).

### Repeat Sequence Analysis

Simple sequence repeats (SSRs) were detected using MISA Perl Script available at http://pgrc.ipk-gatersleben.de/misa/ with the following thresholds: 8 repeat units for mononucleotide SSRs, 4 repeat units for di- and trinucleotide repeat SSRs, and 3 repeat units for tetra-, penta-, and hexanucleotide repeat SSRs. Tandem repeats were analyzed using Tandem Repeats Finder (Benson, 1999) with parameter settings of two for matches and seven for mismatches and indels. The minimum alignment score and maximum period size were set at 50 and 500, respectively. All the identified repeats were manually verified and nested, or redundant results were removed. REPuter (Kurtz et al., 2001) was employed to identify the IRs in *S. cusia* by forward versus reverse complement (palindromic) alignment. The minimal repeat size was set at 30 bp, and the cutoff for similarities among the repeat units was set at 90%.

### Comparative Genome Analysis

Conserved sequences were identified between the cp genomes of *Astragalus membranaceus* and those of *A. paniculata* (NC_022451.2), *R. breedlovei* (KP300014.1), *Tanaecium tetragonolobum* (NC_027955.1), *Dorcoceras hygrometricum* (NC_016468.1), *Salvia miltiorrhiza* (NC_020431.1), *Olea europaea* (NC_013707.2), *Sesamum indicum* (NC_016433.2), and *Scrophularia takesimensis* (NC_026202.1) by using BLASTN with an E-value cutoff of 1e-10. The homologous regions and gene annotations were visualized using a web-based genome synteny viewer GSV (Revanna et al., 2011).

### Comparison of IR Boundaries in the Angiosperm Plant

A total of 31 species were examined for expansion or contraction of the IR, representing 29 species from Lamiales and 2 species from Actinidiaceae. The annotations of these cp genomes were downloaded from RefSeq database.

### Phylogenetic Analysis

A total of 28 complete cp DNA sequences belonging to the Lamiales order were obtained from RefSeq database. For the phylogenetic analysis, 65 protein sequences were shared among all these 31 species, and *S. cusia* was aligned using the CLUSTALW2 (v2.0.12) program. The 65 proteins included ATPA, ATPB, ATPE, ATPF, ATPH, ATPI, CCSA, CEMA, MATK, NDHA, NDHB, NDHC, NDHE, NDHF, NDHG, NDHH, NDHI, NDHJ, NDHK, PETA, PETD, PETG, PETL, PETN, PSAA, PSAB, PSAC, PSAI, PSAJ, PSBA, PSBC, PSBD, PSBE, PSBF, PSBH, PSBJ, PSBK, PSBL, PSBM, PSBN, PSBT, PSBZ, RBCL, RPL14, RPL2, RPL20, RPL22, RPL23, RPL32, RPL33, RPL36, RPOB, RPOC1, RPOC2, RPS11, RPS14, RPS15, RPS18, RPS2, RPS3, RPS7, RPS8, YCF2, YCF3, and YCF4 (Supplementary File 1). The alignment was manually examined and adjusted. Then, the evolutionary history was inferred using the Maximum Likelihood method implemented in RaxML (v8.2.4) (Stamatakis, 2015). The detailed parameters were “raxmlHPC-PTHREADS-SSE3 -f a -N 1000 -m PROTGAMMACPREV -x 551314260 -p 551314260 -o A_thaliana, N_tabacum -T 20”. The tree with the highest log likelihood (-126826.029496) was shown. The significance level for the phylogenetic tree was assessed by bootstrap testing with 1000 replications. The bootstrap values exceeding 50% were shown next to the corresponding nodes.

## Results

### General Features of the *S. cusia* Cp Genome

**FIGURE 1:**
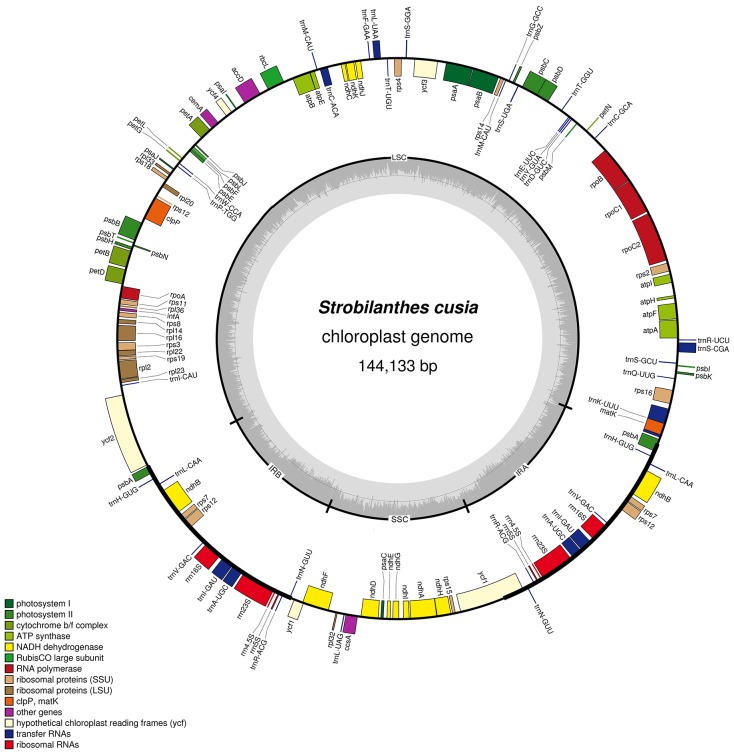
Gene map of *S. cusia* cp genome. Genes inside and outside of the circle are transcribed in the clockwise and counterclockwise directions, respectively. LSC, large single-copy region; SSC small single-copy region; IR, inverted repeat.

The complete plastid genome of *S. cusia* is a circular molecule of 144,133 bp in length. The genome possesses an LSC region of 91,666 bp, separated from the 17,811 bp SSC region by two IRs, each at 17,328 bp (**Figure 1**). The genome was deposited in GenBank under accession number: MG874806. A total of 113 genes are contained within the *S. cusia* cp genome, including 79 protein-coding genes, 30 tRNA genes, and 4 RNA genes (**Table 1**). Five protein-coding and seven tRNA-coding genes are duplicated on the IR regions. The LSC region contains 64 protein-coding genes and 22 tRNA genes, whereas the SSC region contains 11 protein-coding genes and one tRNA gene. Fifteen and three genes in the *S. cusia* cp genome possess one and two introns, respectively. Compared with the genes of *R. breedlovei*, the two genes *matk* and *ycf2* are shorter at 453 and 663 bp, respectively, at the 5′-end sequence. The sequences of *ycf2* and *matk* are confirmed via direct PCR amplifications and Sanger sequencing using the primers listed in Supplementary Table S1.

**Table 1:** Genes contained in the *S. cusia* cp genome (a total of 113 genes).

Category for genes	Group of genes	Name of genes
Ribosome RNA genes	rRNA genes	*rrn16* (IR), *rrn23* (IR), *rrn4.5* (IR), *rrn5* (IR)
Transfer RNA genes	tRNA genes	37 genes (8 contain introns, 7 in the IR regions)
Protein-coding genes	Large subunit of ribosome	*rpl2*^∗^, *rpl14*, *rpl16*^∗^, *rpl20*, *rpl22*, *rpl23*, *rpl32*, *rpl33*, *rpl36*
	Small subunit of ribosome	*rps2*, *rps3*, *rps4*, *rps7* (IR), *rps8*, *rps11*, *rps12* (IR^∗^), *rps14*, *rps15*, *rps16*^∗^,*rps18*, *rps19*
	DNA dependent RNA polymerase	*rpoA*, *rpoB*, *rpoC1*^∗^, *rpoC2*
	Subunits of NADH-dehydrogenase	*ndhA*^∗^, *ndhB*^∗^ (IR), *ndhC*, *ndhD*, *ndhE*, *ndhF*, *ndhG*, *ndhH*, *ndhI*, *ndhJ*, *ndhK*
	Subunits of cytochrome b/f complex	*petA*, *petB*^∗^, *petD*^∗^, *petG*, *petL*, *petN*
	Subunits of photosystem I	*psaA*, *psaB*, *psaC*, *psaI*, *psaJ*, *ycf3*^∗∗^
	Subunits of photosystem II	*psbA* (IR), *psbB*, *psbC*, *psbD*, *psbE*, *psbF*, *psbH*, *psbI*, *psbJ*, *psbK*, *psbL*, *psbM*, *psbN*, *psbT*, *psbZ*
	Subunit of rubisco	*rbcL*
	Subunits of ATP synthase	*atpA*, *atpB*, *atpE*, *atp*F^∗^, *atpH*, *atpI*
	Subunit of Acetyl-CoA-carboxylase	*accD*
	c-type cytochrome synthesis gene	*ccsA*
	Envelop membrane protein	*cemA*
	Protease	*clpP*^∗∗^
	Translational initiation factor	*infA*
	Maturase	*matK*
	Conserved open reading frames	*ycf1* (IR), *ycf2*, *ycf4*


**Genes located on the IR regions are indicated by the (IR) symbol after the gene name. One and two asterisks after gene name reflect one and two introns contained in the gene, respectively. The rps12 transcript is trans-spliced.**

The overall GC content of the *S. cusia* cp genome is 38%. The GC content of the IR regions (46%) is higher than that of the LSC and SSC regions (37% and 32%). Overall, 49.4% of the *S. cusia* cp genome sequence is composed of genes that encode proteins. The overall GC content of the *S. cusia* cp genome is 38.38%, whereas that of the protein-coding regions is 38.00%. Within the protein-coding regions, the GC contents for the first, second, and third positions of codons are 46.17%, 38.51%, and 30.47%, respectively. The codon usage and codon-anticodon recognition patterns of the *S. cusia* cp genome are summarized in Supplementary Table S2. The 30 tRNA genes contain codons corresponding to all 20 amino acids that are necessary for protein biosynthesis.

### Repeat and SSR Analysis

Simple Sequence Repeats are valuable molecular markers of high-degree variations within the same species; these markers have been used in population genetics and polymorphism investigations (Xue et al., 2012). We analyzed the occurrence, type, and distribution of SSRs in the *S. cusia* cp genome. In total, 160 SSRs were identified in *S. cusia* cp genome by using the software tool MISA (**Table 2**). Among these SSRs, the majority consisted of mono- and dinucleotide repeats, which were found 94 and 43 times, respectively. By contrast, tri- (10), tetra- (11), and pentanucleotide repeat sequences (2) were observed with lower frequency. Most mononucleotide repeat sequences consisted of A/T repeats (92.6%). Similarly, 55.8% of the dinucleotide repeat sequences consisted of AT/AT repeats (**Table 2**). These results are in agreement with the previous findings stating that the cp SSRs are generally composed of short poly-A or poly-T repeats and rarely contained tandem G or C repeats (Kuang et al., 2011).

**Table 2:** Numbers of SSRs identified in the cp genome of *S. cusia.*

SSR Repeat Type (Number of copies)	SSR Repeat Sequences	Number of Copies									Total
			
		3	4	5	6	7	8	9	10	11	
Mono-nucleotide (94)	A/T	–	–	–	–	–	45	28	11	3	87
	C/G	–	–	–	–	–	4	2	1		7
Di-nucleotide (43)	AC/GT	–	2								2
	AG/CT	–	15								15
	AT/AT	–	16	4	3	1					24
	CG/CG	–	2								2
Tri-nucleotide (10)	AAG/CTT	–	1								1
	AAT/ATT	–	7	1	1						9
Tetra-nucleotide (11)	AAAG/CTTT	2									2
	AAAT/ATTT	2									2
	AACT/AGTT	1									1
	AATC/ATTG	1									1
	AATG/ATTC	1									1
	AATT/AATT	4									4
Penta-nucleotide (2)	AAAAT/ATTTT	2									2


The locations of SSRs in the cp genome are shown in Supplementary Table S3. The corresponding locations were further classified as intergenic (IGS), exonic, and intronic regions. Several SSRs that were separated with distances less than 100 bp were considered together as a compound SSR. In total, 21 compound SSRs were identified. In terms of the distribution, 64, 46, and 18 repeats were located in the IGS, exon, and intron regions, respectively. In particular, two of them spanned over the IGS and CDS regions (repeats no. 51 and 101, Supplementary Table S3).

By definition, SSRs possess a repeat unit size equal to or less than five. We analyzed repeats with unit sizes exceeding five by using software tool Tandem Repeats Finder and REPuter. The repeats were divided into two types: tandem repeats and dispersed repeats. Tandem repeats are defined as those with two or more adjacent, approximate copies of a pattern of nucleotides. In total, 20 tandem repeats with repeat unit size longer than 12 bp (included) were identified, with the overall percentage of similarities between the adjacent repeat units set to 60% (Supplementary Table S4). The repeat unit was enclosed with parentheses. Among them, 4, 14, and 2 repeats were located in the CDS, intergenic, and intron regions, respectively. In contrast to tandem repeats, dispersed repeats is defined as with two or more approximate copies of a pattern of nucleotides separated by a certain number of nucleotides. Dispersed repeats can be further divided into four types, namely, forward, reverse, complement, and palindromic repeats (Kurtz et al., 2001). In total, two forward repeats and six palindromic repeats were identified using REPuter with a total length cutoff of 30 bp (Supplementary Table S5). For the palindromic repeat, the complementary structures were shown using pairing parentheses. This large set of repeats provides a rich resource for the development of novel molecular markers and to understand the genome evolution in *S. cusia*.

### Genes With Introns

Analysis of the genome revealed a total of 18 genes with introns (**Table 3**). Among the genes, the *rps12* is a trans-spliced gene; its 5′ end is located on the LSC region, and the 3′ end is located on the IR region. Two other genes, *clpP* and *ycf3*, presented two introns and three exons each. Nine genes, namely, *atpF*, *ndhA*, *ndhB*, *petB*, *petD*, *rpl16*, *rpl2*, *rpoC1*, and *rps16*, each with one intron, were identified. Finally, six tRNA genes with one intron and two exons each, namely, *trnA*-UGC, *trnC*-ACA, *trnI*-GAU, *trnK*-UUU, *trnL*-UAA, and *trnS*-CGA, were observed. The lengths of these introns range from 476 bp to 2391 bp, with the longest intron found in the *trnK*-UUU gene.

**Table 3:** Length of intron and exon splitting genes in the *S. cusia* cp genome.

Gene	Length (bp)				
	
	Exon I	Intron I	Exon II	Intron II	Exon III
*rps12^∗^*	114	–	230	536	26
*clpP*	71	736	297	605	229
*ycf3*	124	722	230	703	153
*atpF*	141	700	471		
*ndhA*	553	1092	539		
*ndhB*	870	586	756		
*petB*	6	731	648		
*petD*	8	672	526		
*rpl16*	9	888	402		
*rpl2*	391	644	470		
*rpoC1*	432	847	1620		
*rps16*	40	882	227		
*trnA-*UGC	38	806	35		
*trnC-*ACA	39	575	55		
*trnI-*GAU	37	917	56		
*trnK-*UUU	38	2391	35		
*trnL-*UAA	36	476	49		
*trnS-*CGA	33	685	60		


**^∗^trans-spliced gene.**

### Gene Loss

**FIGURE 2:**
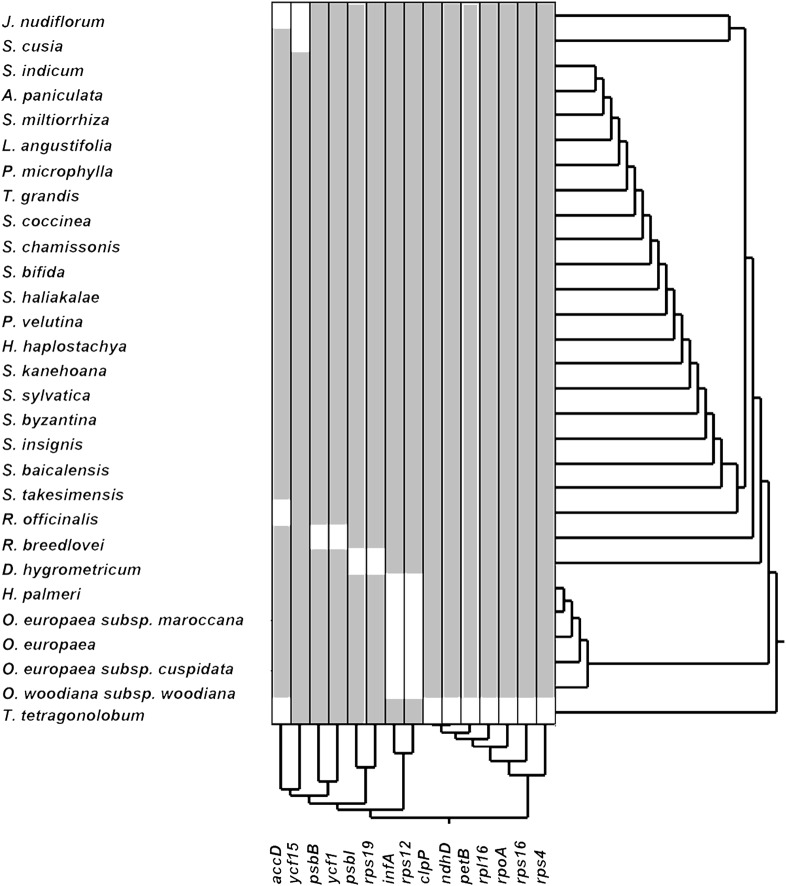
Gene losses in the cp genomes of the Lamiales order.

The loss of genes in cp genomes may result from the transfer of genes into the nuclear genome or the deletion of genes. To understand which genes may become indispensable during evolution, we performed hierarchical clustering of lost genes by using Ward’s method among 29 species in the Lamiales order (**Figure 2**). As shown in **Figure 2**, a total of 15 genes that have lost one gene in at least one species are clustered into five groups. The first group includes genes *accD* and *ycf15*, which are lost in three and two species, respectively. Genes *psbB* and *ycf1*, which are lost in *R. breedlovei*, are included in the second group. Genes *psbI* and *rps19*, which are lost in *D. hygrometricum*, are included in the third group. Genes *rps12* and *infA*, which are included in the fourth group, are lost in five species, including *Hesperelaea palmeri*, three *O. europaea* subsp. *maroccana*, and *Olea woodiana*. The last group includes seven genes, namely, *clpP*, *ndhD*, *petB*, *rpl16*, *rpoA*, *rps16*, and *rps4*, which are all lost in *T. tetragonolobum.* In *S. cusia*, only the *ycf15* gene is lost, indicating that the gene loss rate in *S. cusia* is lower than those in other species. The *ycf15* gene belongs to the PFAM protein family PF10705, in which its function remains unknown. In certain plant species, the *ycf15* gene is probably not a protein-coding gene because the proteins in these species possess premature stop codons (Steane, 2005). The loss of *ycf15* gene results in further undermining of the notion that *ycf15* plays important functions in the genome of *S. cusia*.

### Comparative Analysis of the Cp Genome Rearrangement

**FIGURE 3:**
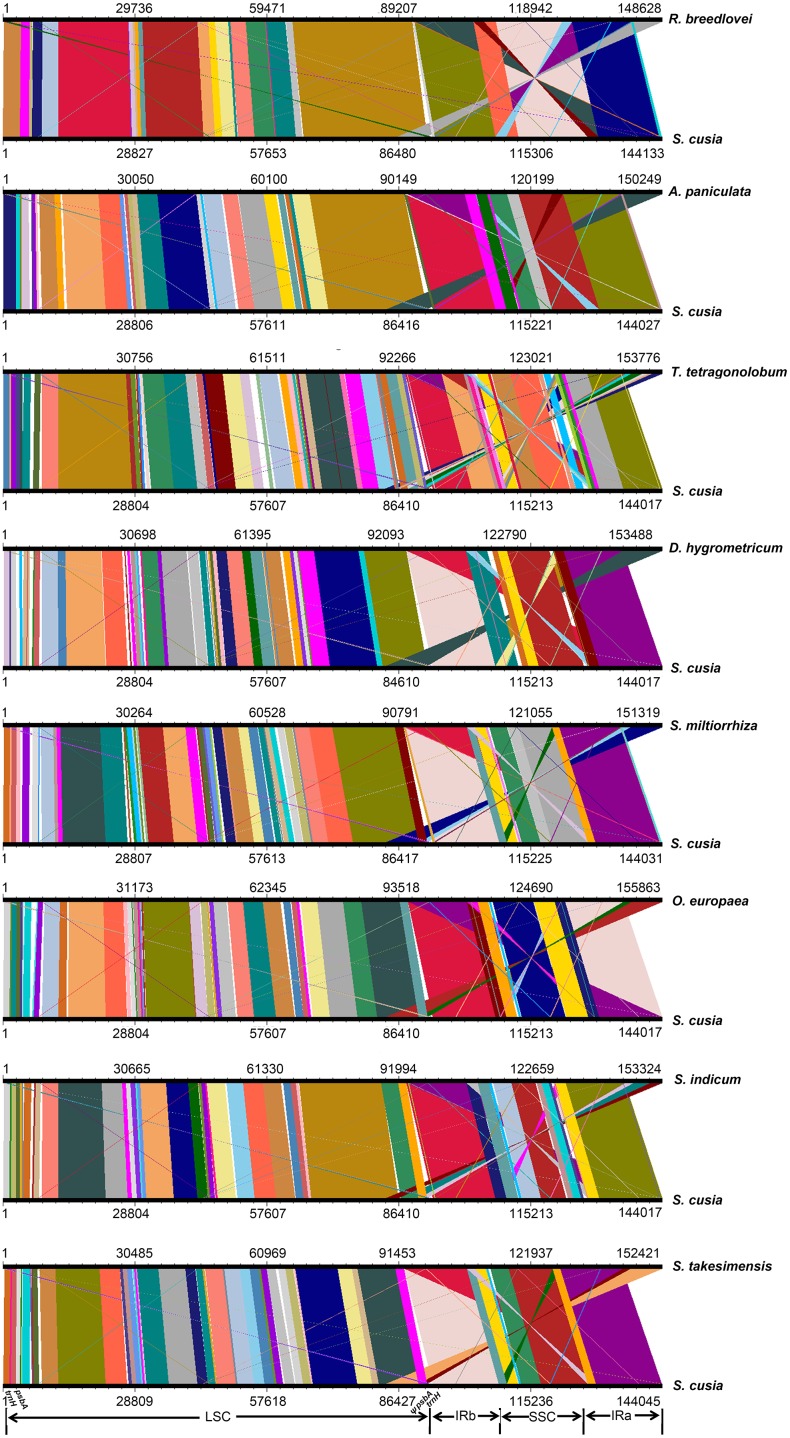
Comparative genomic analyses of *S. cusia* and eight other representative species of Lamiales. The cp genome of *S. cusia* was aligned with those of eight species. Each horizontal black line represents one genome. The species names are shown to the right of the corresponding line. The conserved regions are bridged by lines.

To identify the possible occurrence of genome rearrangement, we selected the cp genome sequences of *S. cusia* and eight other species belonging to Lamiales for synteny analyses. These eight species included *A. paniculata*, *R. breedlovei*, *T. tetragonolobum*, *D. hygrometricum*, *S. miltiorrhiza*, *O. europaea*, *S. indicum*, and *S. takesimensis*, which are members of the Acanthaceae, Bignoniaceae, Gesneriaceae, Lamiaceae, Oleaceae, Pedaliaceae, and Scrophulariaceae families, respectively (**Figure 3**). Members of the Orobanchaceae and Lentibulariaceae families were not included. Orobanchaceae are parasites; without chlorophyll, their cp genomes are considerably reduced both in size and gene content and suffer horizontal gene transfer from its host, gene loss, and gene pseudogenization (Li et al., 2013). An exclusive combination of losses and pseudogenization of the plastid NAD(P)H-dehydrogenase (*ndh*) gene complex was observed in the cp genome of the Lentibulariaceae family (Silva et al., 2016). Therefore, we did not discuss the species belonging to family Lentibulariaceae and Orobanchaceae in the phylogenetic analysis and IR contraction/extension analysis part. Pairwise cp genome comparison between *S. cusia* and the other eight cp genomes recovered a high degree of synteny (**Figure 3**). However, the *S. cusia* cp genome exhibits two unique features: first, the genes of *psbA* and *trnH-*GUG are duplicated in the *S. cusia* cp genome, which are located near the boundaries of the IRa-LSC, and IRb-LSC regions; second, the IR sequence of *S. cusia* is 17,328 bp in length, which is significantly shorter than those of *A. paniculata* (25,340 bp) and *R. breedlovei* (22,704 bp).

### Phylogenetic Analysis of *S. cusia* Based on Conserved Protein Sequences

**FIGURE 4:**
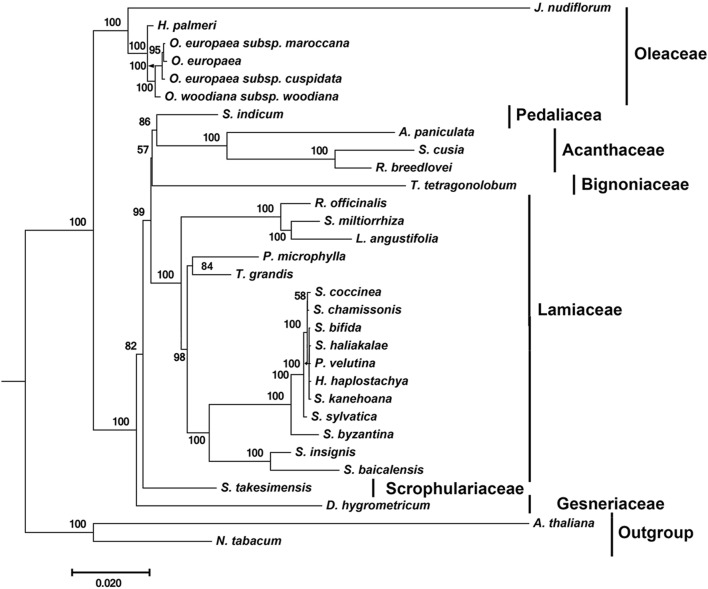
Molecular phylogenetic analyses of cp genomes in the Lamiales order. The tree was constructed with the sequences of 65 proteins present in 31 species (*A. paniculata*, *R. breedlovei*, *T. tetragonolobum*, *D. hygrometricum*, *H. haplostachya*, *L. angustifolia*, *Phyllostegia velutina*, *Premna microphylla*, *R. officinalis*, *S. miltiorrhiza*, *S. baicalensis*, *S. insignis*, *S. byzantina*, *S. chamissonis*, *S. coccinea*, *S. sylvatica*, *S. bifida*, *S. haliakalae*, *S. kanehoana*, *T. grandis*, *H. palmeri*, J. *nudiflorum*, *O. europaea*, *O. europaea* subsp. Cuspidata, *O. europaea* subsp. Maroccana, *O. woodiana* subsp. *woodiana*, *S. indicum*, *S. takesimensis*, *Strobilanthes cusia*, *A. thaliana*, and *N. tabacum*) using the Maximum Likelihood method implemented in RAxML. Two taxa, *N. tabacum* and *A. thaliana*, were used as outgroups. Tribes to which each species belongs are shown to the right side of the tree. Bootstrap supports were calculated from 1000 replicates.

By far, only two cp genomes have been completely sequenced for species from Acanthaceae, a family under the order Lamiales. To determine the phylogenetic position of *S. cusia* in Lamiales, we obtained 29 complete cp genome sequences belonging to the Lamiales from the RefSeq database (Supplementary Table S6). The distributions of the 29 species among different families are as follows: Lamiaceae (16), Bignoniaceae (1), Acanthaceae (3), Gesneriaceae (1), Oleaceae (6), Pedaliaceae (1), and Scrophulariaceae (1), with the number shown in the enclosed parentheses representing the number of species in the corresponding clade. We extracted 65 protein sequences present among the 29 cp genomes and those of *Nicotiana tabacum* and *Arabidopsis thaliana*, which served as the outgroup taxa. Multiple sequence alignment of the amino acid sequences resulted in a total of 19,277 positions in the final dataset. The phylogenetic tree was constructed using the Maximum Likelihood method and is shown in **Figure 4**. The closest sister species of *S. cusia* are *R. breedlovei* and *A. paniculata* in Acanthaceae, (bootstrap values: 100%), and this finding was consistent with the expected relationships.

### IR Contraction/Extension Analysis of the Angiosperms

**FIGURE 5:**
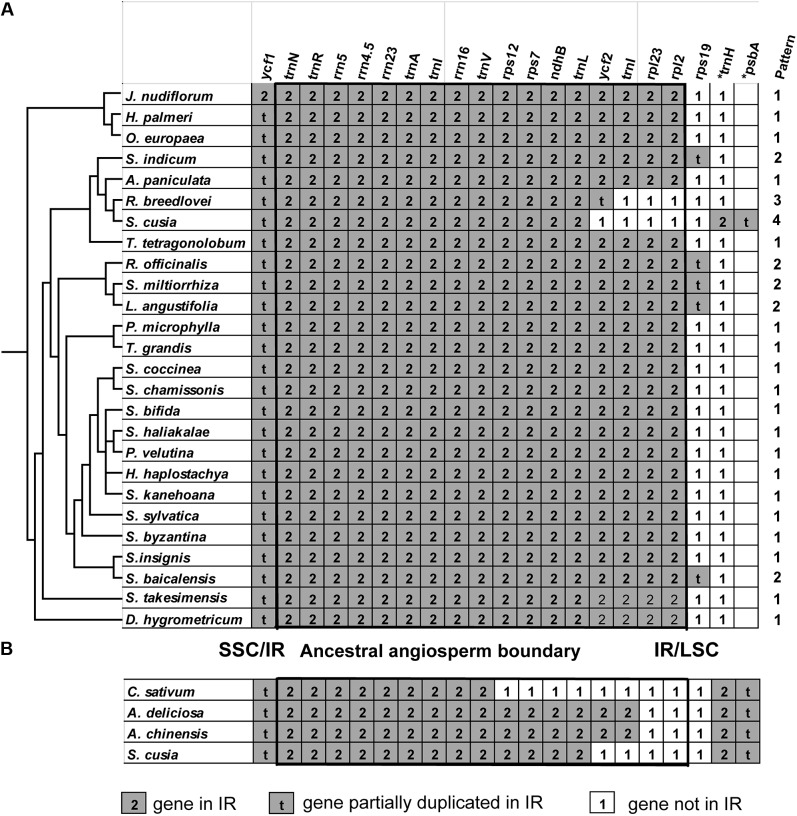
Comparison of IR contents **(A)** between *S. cusia* and several representative cp genomes from Lamiales and **(B)** between *S. cusia* and three non-Lamiales cp genomes. The ancestral angiosperm IR regions are shown in square.

One distinct feature of the *S. cusia* cp genome is its IR length of 17,328 bp, which is significantly shorter than those of most angiosperms [ca. 20–28 kb, (Chumley et al., 2006)]. The shortening of the IR region suggests the occurrence of an IR contraction event. By contrast, the IRs of *S. cusia* spanned to include the *trnH* and *psbA* genes, indicating the occurrence of another IR expansion event. To identify any potential evolutionary events, we analyzed the gene contents at the border areas in detail. The regions around the IR-LSC and IR-SSC borders extending from all the available cp genomes in Lamiales were analyzed, except those from the family Lentibulariaceae (the bladderwort family) and Orobanchaceae (a family of parasitic plants) (**Figure 5A**). *S. cusia* is the only species in the Lamiales with an SSC region containing two copies of the *trn*H genes and two partial *psb*A genes. To determine the uniqueness of this pattern, we searched the SSC regions of all cp genomes and identified three species, *Coriandrum sativum*, *Actinidia deliciosa*, and *Actinidia chinensis*, whose SSC regions contain two copies of the *trn*H and *psbA* genes (**Figure 5B**).

The genes at the IR/SSC regions are rather conserved (**Figure 5**), given that *ndhF* (not shown in the alignment) and *ycf1* genes are found in the SSC regions near the borders in almost all examined cp genomes examined; both genes appeared on the left side of the alignment in **Figures 5A,B**. By contrast, various patterns of expansion and contraction of the IR regions into or from the LSC regions were observed, as shown on the right side of the alignment in **Figures 5A,B**.

LSC/IR regions can be divided into four different types based on the patterns for the 29 cp genomes (**Figure 5A**) belonging to the Lamiales. Type I, the most dominant pattern, is found in 22 species, including *D. hygrometricum*, *T. tetragonolobum*, *S. takesimensis*, *A. paniculata*, *Scutellaria insignis*, *Stenogyne kanehoana*, *Stenogyne haliakalae*, *Stenogyne bifida*, *Phyllostegia velutina*, *Haplostachys haplostachya*, *Stachys chamissonis*, *Stachys byzantina*, *Stachys coccinea*, *Stachys sylvatica*, *Premna microphylla*, *Tectona grandis*, *Jasminum nudiflorum*, *H. palmeri*, *O. woodiana* subsp. *woodiana*, *O. europaea*, *O. europaea* subsp. *maroccana*, and *O. europaea* subsp. *cuspidate*. For this type, the genes included *rpl2*, *rpl23*, *trnI*, *ycf2, trnL, ndhB*, *rps7*, *3-rps12*, *trnV*, *rrn16*, *trnI*, *trnA*, *rrn23*, *rrn4.5*, *rrn5*, *trnR*, *trnN*, and *ycf1* (pseudogene), which are duplicated in the IR regions, except for *rps19*. The LSC/IR border patterns of *O. woodiana* subsp. *woodiana*, *O. europaea* subsp. *maroccana*, and *O. europaea* subsp. *cuspidata* are the same as that of *O. europaea*. As a result, their structures of the LSC/IR regions are not shown in **Figure 5A**.

The Type II pattern is found in five species, including *Scutellaria baicalensis*, *S. miltiorrhiza*, *Rosmarinus officinalis*, *Lavandula angustifolia*, and *S. indicum*. This pattern shows an *rps19* pseudogene at the IR/LSC border. Meanwhile, Type III pattern is only found in *R. breedlovei*, in which the *rps19, rpl2*, and *rpl22* are not found in the IR region, and an *ycf2* pseudogene is found at the IR/LSC border.

Type IV is found only in the *S. cusia* cp genome. On the one hand, the genes of *rps19*, *rpl2*, *rpl23*, and *ycf2* are found in the IRs. On the other hand, the IR extends into the LSC regions to include a *trnH* gene and a truncated *psbA* pseudogene at the IR/LSC border. A similar pattern has been observed in several species other than Lamiales (**Figure 5B**). For example, in the cp genomes of *C. sativum* and *A. chinensis*, the IR region extends into the *psbA* gene and engulfs a fragment of the *psb*A gene at the IR/LSC border. However, these patterns slightly vary. In the cp genome of *C. sativum*, a serial of genes, including *rps19*, *rpl2*, *rpl23*, *ycf2*, *ndhB*, *rps7*, and *3-rps12*, are excluded from the IR regions. Meanwhile, in *A. chinensis*, the IR-LSC border expanded to the *ycf2* gene. Compared with the cp genome of other Lamiales, the loss of genes *rpl2*, *rpl23*, and *ycf2* in the IR regions may be responsible for the considerably shortened length of *S. cusia* cp genome. The various patterns of the IR/LSC border reflect the highly dynamic nature of the IR/LSC border regions.

## Discussion

We carried out a detailed study of the cp genome of *S. cusia*. In particular, we (1) sequenced the total DNA from the leaves of *S. cusia*, (2) filtered the reads that are similar to cp genome of other species, (3) assembled the genome followed by gap-filling to obtain the complete cp genome, (4) annotated the genome to identify protein-coding genes, rRNAs, and tRNAs, (5) analyzed the repeat sequences, (6) identified multiple SSR sequences, (7) characterized the boundary of the IR regions, and (8) conducted phylogenetic analyses of *S. cusia* and several closely related species. The present study provides high-quality reference sequences that can be used to precisely determine the varieties of *S. cusia*.

With the widespread adoption of next-generation DNA sequencing technology and bioinformatics software tools, complete plastid genome sequencing becomes possible with the genome skimming approach (Twyford and Ness, 2016). In the current study and several of our previous studies (Chen et al., 2014; Wang et al., 2016), the use of Illumina and PacBio technologies can lead to the assembly of the complete cp genome without the tedious isolation of plastids to separate cp DNA from nuclear DNAs. However, the polyploidy nature of the plastid genome makes the heteroplasmic variations almost a rule rather than exception (Lei et al., 2016). Thus, the genome sequence reported here only represents the most predominant types, and the diversities among the various cp genomes may have been neglected. One future challenge is to accurately characterize the variations in plastid genomes to understand the evolution of thousands of copies of plastid genome within a particular cell, within an individual, or within a particular species.

Ban-Lan-Gen is considered a common name for the dried roots of indigo plants; among them, *I. indigotica* Fort and *B. cusia* (Nees) Bremek are the most frequently used plants. Pharmacological studies have confirmed the anti-inflammatory and anti-viral effects of Ban-Lan-Gen for the treatment of hepatitis, influenza, and various kinds of inflammation (Sun et al., 2008; Gu et al., 2014). Studies on the chemical components have identified several biologically active ingredients in Ban-Lan-Gen, including indigoid alkaloids (such as indigo and indirubin) and quinazolinone alkaloids (such as tryptanthrin) (Ichimaru et al., 2015). Indigo, indirubin, and tryptanthrin are three marker compounds found in Ban-Lan-Gen (Merz et al., 2004), and both *I. indigotica* Fort and *B. cusia* (Nees) Bremek contain these compounds. Meanwhile, indirubin causes the transactivation of cytochrome P450 1A1 (CYP1A1) and CYP1A2 genes via the aryl hydrocarbon receptor (Otsuka et al., 1988). These studies have demonstrated the effectiveness of Ban-Lan-Gen in the treatment of various diseases and presented concerns whether the biological components from various plant materials are identical. For ensuring consistent results from investigations and the efficacy and effective usage of any medicinal product with Ban-Lan-Gen as their source materials, the exact identity of the plant material should be determined. We carried out the sequencing and analysis of the complete genome of *S. cusia* to solve this problem.

The cp genome of *S. cusia* is the first cp genome in the genera *Strobilanthes* (Strobilanthinae sensu Bremekamp). Roughly 400 species were recorded in tropical Asia. Specifically, 128 species, including 57 endemic ones, were discovered in China (Flora of China Editorial Committee, 2011). Species belonging to this genera display significant high levels of homoplasy in morphological characters, thereby causing marked difficulty in subdividing the genus. By contrast, molecular studies do not improve the situation because of insufficient sampling. Even the available samples appear to be challenging because numerous species are known only from the type of collection or from materials that are inadequate for molecular study. Taking these results together, we find that although the clusters of related species are clearly discernible, sufficient information is currently unavailable to produce a satisfactory intra-generic classification. As a result, the acquisition of the first cp genome opens up the avenue for molecular classification of species in the genera.

As a proof of concept exercise, we designed a set of 1563 primers using the program ecoPrimer^2^. They were identified using the following parameters: “-l 299 -L 301 -e 0 -t species” and the detailed results are shown in Supplementary File S1. These primers can be used to distinguish *S. cusia* and three of its most closely related species: *A. paniculata*, *R. breedlovei* and *T. tetragonolobum*. In addition, we designed a set of primers that can be used to amplify 130 SSR sequences shown in Supplementary Table S3 using the program primer3^3^. The detailed results are shown in Supplementary File S2. The validation of these primers for effective inter-specific and intra-specific discrimination will be the subject of future studies.

The cp genomes offer numerous applications. First, the cp genome is rich with molecular markers that can be used to distinguish different species (Song et al., 2015; Yang et al., 2016; Xu et al., 2017); different varieties from the same species (Xu et al., 2015; Pervaiz et al., 2016), and possibly different individuals within the same species (Lei et al., 2016). Owing to the acquisition of the first complete genome of *S. cusia*, sequencing of additional plastid genomes from other *Strobilanthes* species and varieties of *S. cusia* becomes increasingly easy, consequently providing the basis for the identification of molecular markers that can be used for identity determination at various levels. Second, the overall high conservation levels of the cp genome are useful for phylogenomic study. Third, plastid genomes have been implicated in genetic engineering to encode and express endogenous and exogenous proteins for health and economic benefits (Daniell et al., 2016; Dyo and Purton, 2018). Identification of the complete genome is a requirement for determining the appropriate insertion sites and designing the primers used in genetic engineering experiments. The results obtained from this study have laid the foundation for future cp-based genetic engineering of *S. cusia*.

The presence of two IRs is one of the most notable features in the cp genomes (cpDNAs). In general, the gene arrangement in IR regions in monocot genomes is different from that in eudicot genomes. For example, in monocots, the *trnH* gene is located in the IR region, and in most eudicots, *trnH* gene is located in the LSC region (Huotari and Korpelainen, 2012). In land plants, dynamic expansion/contraction of IRs has been previously reported in several lineages, such as Berberidaceae (Ma et al., 2013), Mimosoid legume (Dugas et al., 2015), Geraniaceae (Weng et al., 2014), and Hydatellaceae (Goremykin et al., 2013), which presents extreme expansions of IRs, Lauraceae (Song et al., 2015), and Apioideae (Plunkett and Downie, 2000), which exhibits extreme contractions of IRs. The fluctuating lengths of IRs contribute to increase or decrease cpDNA sizes and can be utilized to address phylogeny issues (Wolf et al., 2010).

## Author Contributions

WW collected the sample of *Strobilanthes cusia* (Nees) Kuntze and wrote part of the Introduction and Discussion sections. CL conceived the study and wrote the Introduction and Discussion sections. ZZ and LH identified the plant material. JS extracted DNA. HC performed data analysis and wrote the manuscript. HZ, MJ, and DY performed the PCR experiments. MH, MR, XL, and BS performed the next-generation sequencing experiments.

## Conflict of Interest Statement

MH, MR, XL, and BS are employees of Illumina Corporation. The other authors declare that the research was conducted in the absence of any commercial or financial relationships that could be construed as a potential conflict of interest.

## Footnotes

1 http://foc.eflora.cn/

2 http://metabarcoding.org/obitools/doc/scripts/ecoPrimers.html

3 https://sourceforge.net/projects/primer3/

## References

[B1] Benson G. (1999). Tandem repeats finder: a program to analyze DNA sequences. *Nucleic Acids Res.* 27 573–580. 10.1093/nar/27.2.573 9862982 PMC148217

[B2] Chen H. Zhang J. Yuan G. Liu C. (2014). Complex interplay among DNA modification, noncoding RNA expression and protein-coding RNA expression in *Salvia miltiorrhiza* chloroplast genome. *PLoS One* 9:e99314. 10.1371/journal.pone.0099314 24914614 PMC4051680

[B3] Chinese Pharmacopoeia Commission (2015). *The Pharmacopoeia of the People’s Republic of China, 2015 Edition Part I.* Beijing: China Medical Science Press.

[B4] Chumley T. W. Palmer J. D. Mower J. P. Fourcade H. M. Calie P. J. Boore J. L. (2006). The complete chloroplast genome sequence of *Pelargonium x hortorum*: organization and evolution of the largest and most highly rearranged chloroplast genome of land plants. *Mol. Biol. Evol.* 23 2175–2190. 10.1093/molbev/msl089 16916942

[B5] Cui L. Veeraraghavan N. Richter A. Wall K. Jansen R. K. Leebens-Mack J. (2006). ChloroplastDB: the chloroplast genome database. *Nucleic Acids Res.* 34 D692–D696. 10.1093/nar/gkj055 16381961 PMC1347418

[B6] Daniell H. Lin C. S. Yu M. Chang W. J. (2016). Chloroplast genomes: diversity, evolution, and applications in genetic engineering. *Genome Biol.* 17:134. 10.1186/s13059-016-1004-2 27339192 PMC4918201

[B7] Dugas D. V. Hernandez D. Koenen E. J. Schwarz E. Straub S. Hughes C. E. (2015). Mimosoid legume plastome evolution: IR expansion, tandem repeat expansions, and accelerated rate of evolution in clpP. *Sci. Rep.* 5:16958. 10.1038/srep16958 26592928 PMC4655330

[B8] Dyo Y. M. Purton S. (2018). The algal chloroplast as a synthetic biology platform for production of therapeutic proteins. *Microbiology* 164 113–121. 10.1099/mic.0.000599 29297850

[B9] Flora of China Editorial Committee (ed.) (2011). *Flora of China.* Beijing: Science Press.

[B10] Golden J. L. Bain J. F. (2000). Phylogeographic patterns and high levels of chloroplast DNA diversity in four Packera (asteraceae) species in southwestern Alberta. *Evolution* 54 1566–1579. 10.1111/j.0014-3820.2000.tb00702.x 11108585

[B11] Goremykin V. V. Nikiforova S. V. Biggs P. J. Zhong B. Delange P. Martin W. (2013). The evolutionary root of flowering plants. *Syst. Biol.* 62 50–61. 10.1093/sysbio/sys070 22851550

[B12] Gu W. Zhang Y. Hao X. J. Yang F. M. Sun Q. Y. Morris-Natschke S. L. (2014). Indole alkaloid glycosides from the aerial parts of *Strobilanthes cusia*. *J. Nat. Prod.* 77 2590–2594. 10.1021/np5003274 25427242

[B13] Honda G. Tabata M. (1979). Isolation of antifungal principle tryptanthrin, from *Strobilanthes cusia* O. Kuntze. *Planta Med.* 36 85–90. 10.1055/s-0028-1097245 461559

[B14] Huotari T. Korpelainen H. (2012). Complete chloroplast genome sequence of *Elodea canadensis* and comparative analyses with other monocot plastid genomes. *Gene* 508 96–105. 10.1016/j.gene.2012.07.020 22841789

[B15] Ichimaru Y. Saito H. Uchiyama T. Metori K. Tabata K. Suzuki T. (2015). Indirubin 3′-(O-oxiran-2-ylmethyl)oxime: a novel anticancer agent. *Bioorg. Med. Chem. Lett.* 25 1403–1406. 10.1016/j.bmcl.2015.02.053 25765906

[B16] Jansen R. K. Cai Z. Raubeson L. A. Daniell H. Depamphilis C. W. Leebens-Mack J. (2007). Analysis of 81 genes from 64 plastid genomes resolves relationships in angiosperms and identifies genome-scale evolutionary patterns. *Proc. Natl. Acad. Sci. U.S.A.* 104 19369–19374. 10.1073/pnas.0709121104 18048330 PMC2148296

[B17] Krumsiek J. Arnold R. Rattei T. (2007). Gepard: a rapid and sensitive tool for creating dotplots on genome scale. *Bioinformatics* 23 1026–1028. 10.1093/bioinformatics/btm039 17309896

[B18] Kuang D. Y. Wu H. Wang Y. L. Gao L. M. Zhang S. Z. Lu L. (2011). Complete chloroplast genome sequence of *Magnolia kwangsiensis* (Magnoliaceae): implication for DNA barcoding and population genetics. *Genome* 54 663–673. 10.1139/G11-026 21793699

[B19] Kumagai T. Aratsu Y. Sugawara R. Sasaki T. Miyairi S. Nagata K. (2016). Indirubin, a component of Ban-Lan-Gen, activates CYP3A4 gene transcription through the human pregnane X receptor. *Drug Metab. Pharmacokinet.* 31 139–145. 10.1016/j.dmpk.2016.01.002 26987505

[B20] Kurtz S. Choudhuri J. V. Ohlebusch E. Schleiermacher C. Stoye J. Giegerich R. (2001). REPuter: the manifold applications of repeat analysis on a genomic scale. *Nucleic Acids Res.* 29 4633–4642. 10.1093/nar/29.22.4633 11713313 PMC92531

[B21] Laslett D. Canback B. (2004). ARAGORN, a program to detect tRNA genes and tmRNA genes in nucleotide sequences. *Nucleic Acids Res.* 32 11–16. 10.1093/nar/gkh152 14704338 PMC373265

[B22] Lee E. Harris N. Gibson M. Chetty R. Lewis S. (2009). Apollo: a community resource for genome annotation editing. *Bioinformatics* 25 1836–1837. 10.1093/bioinformatics/btp314 19439563 PMC2705230

[B23] Lei W. Ni D. Wang Y. Shao J. Wang X. Yang D. (2016). Intraspecific and heteroplasmic variations, gene losses and inversions in the chloroplast genome of *Astragalus membranaceus*. *Sci. Rep.* 6:21669. 10.1038/srep21669 26899134 PMC4761949

[B24] Leister D. (2003). Chloroplast research in the genomic age. *Trends Genet.* 19 47–56. 10.1016/S0168-9525(02)00003-3 12493248

[B25] Li X. Zhang T. C. Qiao Q. Ren Z. Zhao J. Yonezawa T. (2013). Complete chloroplast genome sequence of holoparasite *Cistanche deserticola* (Orobanchaceae) reveals gene loss and horizontal gene transfer from its host *Haloxylon ammodendron* (Chenopodiaceae). *PLoS One* 8:e58747. 10.1371/journal.pone.0058747 23554920 PMC3598846

[B26] Liu C. Shi L. Zhu Y. Chen H. Zhang J. Lin X. (2012). CpGAVAS, an integrated web server for the annotation, visualization, analysis, and GenBank submission of completely sequenced chloroplast genome sequences. *BMC Genomics* 13:715. 10.1186/1471-2164-13-715 23256920 PMC3543216

[B27] Liu Y. Ahmed S. Liu B. Guo Z. Huang W. Wu X. (2014). Ethnobotany of dye plants in Dong communities of China. *J. Ethnobiol. Ethnomed.* 10:23. 10.1186/1746-4269-10-23 24552267 PMC3998736

[B28] Lohse M. Drechsel O. Kahlau S. Bock R. (2013). OrganellarGenomeDRAW–a suite of tools for generating physical maps of plastid and mitochondrial genomes and visualizing expression data sets. *Nucleic Acids Res.* 41 W575–W581. 10.1093/nar/gkt289 23609545 PMC3692101

[B29] Lowe T. M. Eddy S. R. (1997). tRNAscan-SE: a program for improved detection of transfer RNA genes in genomic sequence. *Nucleic Acids Res.* 25 955–964. 10.1093/nar/25.5.0955 9023104 PMC146525

[B30] Ma J. Yang B. Zhu W. Sun L. Tian J. Wang X. (2013). The complete chloroplast genome sequence of *Mahonia bealei* (Berberidaceae) reveals a significant expansion of the inverted repeat and phylogenetic relationship with other angiosperms. *Gene* 528 120–131. 10.1016/j.gene.2013.07.037 23900198

[B31] Merz K. H. Schwahn S. Hippe F. Muhlbeyer S. Jakobs S. Eisenbrand G. (2004). Novel indirubin derivatives, promising anti-tumor agents inhibiting cyclin-dependent kinases. *Int. J. Clin. Pharmacol. Ther.* 42 656–658. 10.5414/CPP42656 15598038

[B32] Nock C. J. Waters D. L. Edwards M. A. Bowen S. G. Rice N. Cordeiro G. M. (2011). Chloroplast genome sequences from total DNA for plant identification. *Plant Biotechnol. J.* 9 328–333. 10.1111/j.1467-7652.2010.00558.x 20796245

[B33] Otsuka H. Hirai Y. Nagao T. Yamasaki K. (1988). Anti-inflammatory activity of benzoxazinoids from roots of *Coix lacryma-jobi* var. ma-yuen. *J. Nat. Prod.* 51 74–79. 10.1021/np50055a009 2453615

[B34] Palme A. E. Su Q. Palsson S. Lascoux M. (2004). Extensive sharing of chloroplast haplotypes among European birches indicates hybridization among *Betula pendula*, *B. pubescens* and *B. nana*. *Mol. Ecol.* 13 167–178. 10.1046/j.1365-294X.2003.02034.x 14653797

[B35] Pervaiz T. Cheng Z. Faheem M. Fang J. (2016). Chloroplast based genetic diversity among Chinese grapes genotypes. *Mitochondrial DNA A DNA Mapp. Seq. Anal.* 28 565–569. 10.3109/24701394.2016.1155119 27159719

[B36] Plunkett G. M. Downie S. R. (2000). Expansion and contraction of the chloroplast inverted repeat in Apiaceae subfamily Apioideae. *Syst. Bot.* 25 648–667. 10.2307/2666726

[B37] Revanna K. V. Chiu C. C. Bierschank E. Dong Q. (2011). GSV: a web-based genome synteny viewer for customized data. *BMC Bioinformatics* 12:316. 10.1186/1471-2105-12-316 21810250 PMC3199762

[B38] Rice P. Longden I. Bleasby A. (2000). EMBOSS: the European molecular biology open software suite. *Trends Genet.* 16 276–277. 10.1016/S0168-9525(00)02024-2 10827456

[B39] Silva S. R. Diaz Y. C. Penha H. A. Pinheiro D. G. Fernandes C. C. Miranda V. F. (2016). The chloroplast genome of *Utricularia reniformis* sheds light on the evolution of the ndh gene complex of terrestrial carnivorous plants from the Lentibulariaceae family. *PLoS One* 11:e0165176. 10.1371/journal.pone.0165176 27764252 PMC5072713

[B40] Simpson J. T. Wong K. Jackman S. D. Schein J. E. Jones S. J. Birol I. (2009). ABySS: a parallel assembler for short read sequence data. *Genome Res.* 19 1117–1123. 10.1101/gr.089532.108 19251739 PMC2694472

[B41] Song Y. Dong W. Liu B. Xu C. Yao X. Gao J. (2015). Comparative analysis of complete chloroplast genome sequences of two tropical trees *Machilus yunnanensis* and *Machilus balansae* in the family Lauraceae. *Front. Plant Sci.* 6:662. 10.3389/fpls.2015.00662 26379689 PMC4548089

[B42] Sun X. B. Sheng J. R. Wang D. P. (2008). Research progress of chemical constituents and pharmacological activities for *Baphicacanthus cusia* (Nees)Bremek. *Guangxi Shifan Xueyuan Xuebao* 25 66–69.

[B43] Stamatakis A. (2015). Using RAxML to infer phylogenies. *Curr. Protoc. Bioinformatics* 51 6.14.1–16.14.14. 10.1002/0471250953.bi0614s51 26334924

[B44] Steane D. A. (2005). Complete nucleotide sequence of the chloroplast genome from the Tasmanian blue gum, *Eucalyptus globulus* (Myrtaceae). *DNA Res.* 12 215–220. 10.1093/dnares/dsi006 16303753

[B45] Twyford A. D. Ness R. W. (2016). Strategies for complete plastid genome sequencing. *Mol. Ecol. Resour.* 17 858–868. 10.1111/1755-0998.12626 27790830 PMC6849563

[B46] Wang B. Chen H. Ma H. Zhang H. Lei W. Wu W. (2016). Complete plastid genome of *Astragalus membranaceus* (Fisch.) Bunge var. membranaceus. *Mitochondrial DNA B Resour.* 1 517–519. 33473540 10.1080/23802359.2016.1197057 PMC7800798

[B47] Weng M. L. Blazier J. C. Govindu M. Jansen R. K. (2014). Reconstruction of the ancestral plastid genome in Geraniaceae reveals a correlation between genome rearrangements, repeats, and nucleotide substitution rates. *Mol. Biol. Evol.* 31 645–659. 10.1093/molbev/mst257 24336877

[B48] Wicke S. Muller K. F. Depamphilis C. W. Quandt D. Bellot S. Schneeweiss G. M. (2016). Mechanistic model of evolutionary rate variation en route to a nonphotosynthetic lifestyle in plants. *Proc. Natl. Acad. Sci. U.S.A.* 113 9045–9050. 10.1073/pnas.1607576113 27450087 PMC4987836

[B49] Wicke S. Schneeweiss G. M. Depamphilis C. W. Muller K. F. Quandt D. (2011). The evolution of the plastid chromosome in land plants: gene content, gene order, gene function. *Plant Mol. Biol.* 76 273–297. 10.1007/s11103-011-9762-4 21424877 PMC3104136

[B50] Wolf P. G. Roper J. M. Duffy A. M. (2010). The evolution of chloroplast genome structure in ferns. *Genome* 53 731–738. 10.1139/g10-061 20924422

[B51] Wu Z. Y. Raven P. H. Hong D. Y. (2006). *Flora of China*. Beijing: Science Press.

[B52] Xu C. Dong W. Li W. Lu Y. Xie X. Jin X. (2017). Comparative analysis of six *Lagerstroemia* complete chloroplast genomes. *Front. Plant Sci.* 8:15. 10.3389/fpls.2017.00015 28154574 PMC5243828

[B53] Xu M. Xu L. A. Cao F. L. Zhang H. J. Yu F. X. (2015). Development of novel chloroplast microsatellite markers for *Ginkgo biloba*. *Genet. Mol. Res.* 14 7715–7720. 10.4238/2015.July.13.17 26214452

[B54] Xue J. Wang S. Zhou S. L. (2012). Polymorphic chloroplast microsatellite loci in Nelumbo (Nelumbonaceae). *Am. J. Bot.* 99 e240–e244. 10.3732/ajb.1100547 22615305

[B55] Yang Y. Zhou T. Duan D. Yang J. Feng L. Zhao G. (2016). Comparative analysis of the complete chloroplast genomes of five *Quercus* species. *Front. Plant Sci.* 7:959. 10.3389/fpls.2016.00959 27446185 PMC4923075

