# Supplementary material for: Sequencing and Analysis of Strobilanthes cusia (Nees) Kuntze Chloroplast Genome Revealed the Rare Simultaneous Contraction and Expansion of the Inverted Repeat Region in Angiosperm
Source: Front Plant Sci. 2018 Mar 14;9:324. doi: 10.3389/fpls.2018.00324 (PMC5861152; doi:10.3389/fpls.2018.00324)
Supplement: Supplementary file 1 [file Data_Sheet_1.zip › Data Sheet 1/1SupplementaryTables.DOCX]

**Title: Sequencing and analysis of *Strobilanthes cusia* (Nees) Kuntze chloroplast genome revealed the rare simultaneous contraction and expansion of the inverted repeat region in angiosperm**

Haimei Chen^1#^, Junjie Shao^1#^, Hui Zhang^1^, Mei jiang^1^, Linfang Huang^1^, Zhao Zhang^1^, Dan Yang^1^, Molly He^3^, Mostafa Ronaghi^3^, Xi Luo^3^, Botao Sun^3^, Wuwei Wu^2^, and Chang Liu*^1^

^1^Key Laboratory of Bioactive Substances and Resource Utilization of Chinese Herbal Medicine from Ministry of Education, Institute of Medicinal Plant Development, Chinese Academy of Medical Sciences and Peking Union Medical College, Beijing, 100193, China

^2^Guangxi Botanical Garden of Medicinal Plants, Nanning, Guangxi 530023, P. R. China

^3^Illumina, Inc., 5200 Illumina Way, San Diego, CA 92122 USA

^#^contributed equally

*To whom correspondence should be addressed. Tel: +86-10-57833111; Fax: +86-10-62899715; Email: [cliu6688@yahoo.com](mailto:cliu6688@yahoo.com)

Email Address：

HMC: hmchen@implad.ac.cn

JJS: shaojie415@126.com

HZ: 18238801021@163.com

MJ: mjiang0502@163.com

LFH: lfhuang@implad.ac.cn

ZZ: zzhang@implad.ac.cn

DY: dyang@implad.ac.cn

MH: mhe@illumina.com

MR: mronaghi@illumina.com

XL: xluo@illumina.com

BTS: bsun@illumina.com

WWW: wuweiwu2013@163.com

CL: cliu6688@yahoo.com; cliu@implad.ac.cn

**Table S1 Primers used for the validation of the genome assembly.**

| ID | Sequence (5' to 3') | Region | Position | PCR Product Size (bp) |
| --- | --- | --- | --- | --- |
| jm.6-1F | GGAATCTTGGCGATCTTCTCTATC | IRa_LSC | 143522-143545 | 2148 |
| jm.6-1R | GGTAACCCACAGCGAAATTCTA | IRa_LSC | 1515-1536 |  |
| jm.7-1F | CAGAGTGCAACCATGCAGTA | IRb_LSC | 92116-92135 | 2163 |
| jm.7-1R | GGAATCTTGGCGATCTTCTCTATC | IRb_LSC | 94255-94278 |  |
| jm_8-1F | CGCCTCTGCATCTAGCATTG | IRb_SSC | 109456-109475 | 964 |
| jm_8-1R | AGAGGATTCGACGCATTGGT | IRb_SSC | 110400-110419 |  |
| jm_9-1F | TGACATCTCGTATCTCACAGCC | IRa_SSC | 127488-127509 | 858 |
| jm_9-1R | GCGCCTCTGCATCTAGCATT | IRa_SSC | 128326-128345 |  |
| jm_10-1F | AACCTCCAGAAGATGTTGATCGT | matK | 2563-2585 | 748 |
| jm_10-1R | AGTCGAAGTTCAAGTGGTGGAA | matK | 3331-3310 |  |
| jm_11-2F | ACTTTCGCGCAATTCACAGG | ycf2 | 86014-86033 | 811 |
| jm_11-2R | TCCGATTCGTGCGGATTCTT | ycf2 | 86843-86824 |  |

**Table S2 The codon-anticodon recognition pattern and codon usage for *S. cusia.***

| Codon | Amino acid | Fraction | Frequency | No. | tRNA | Codon | Amino acid | Fraction | Frequency | No. | tRNA |
| --- | --- | --- | --- | --- | --- | --- | --- | --- | --- | --- | --- |
| GCA | A | 0.278 | 15.746 | 374 | trnA-UGC | AAT | N | 0.756 | 34.523 | 820 |  |
| GCC | A | 0.17 | 9.641 | 229 |  | CCA | P | 0.276 | 11.578 | 275 | trnP-TGG |
| GCG | A | 0.112 | 6.357 | 151 |  | CCC | P | 0.209 | 8.757 | 208 |  |
| GCT | A | 0.44 | 24.966 | 593 |  | CCG | P | 0.142 | 5.936 | 141 |  |
| TGC | C | 0.241 | 2.61 | 62 | trnC-GCA | CCT | P | 0.373 | 15.662 | 372 |  |
| TGT | C | 0.759 | 8.21 | 195 | trnC-ACA | CAA | Q | 0.765 | 26.903 | 639 | trnQ-UUG |
| GAC | D | 0.196 | 7.705 | 183 | trnD-GUC | CAG | Q | 0.235 | 8.252 | 196 |  |
| GAT | D | 0.804 | 31.576 | 750 |  | AGA | R | 0.298 | 17.767 | 422 | trnR-UCU |
| GAA | E | 0.745 | 37.849 | 899 | trnE-UUC | AGG | R | 0.1 | 5.978 | 142 |  |
| GAG | E | 0.255 | 12.967 | 308 |  | CGA | R | 0.237 | 14.146 | 336 |  |
| TTC | F | 0.341 | 19.914 | 473 | trnF-GAA | CGC | R | 0.072 | 4.294 | 102 |  |
| TTT | F | 0.659 | 38.481 | 914 |  | CGG | R | 0.082 | 4.884 | 116 |  |
| GGA | G | 0.383 | 26.987 | 641 |  | CGT | R | 0.21 | 12.546 | 298 | trnR-ACG |
| GGC | G | 0.101 | 7.115 | 169 | trnG-GCC | AGC | S | 0.063 | 4.8 | 114 | trnS-GCU |
| GGG | G | 0.186 | 13.136 | 312 |  | AGT | S | 0.199 | 15.115 | 359 |  |
| GGT | G | 0.33 | 23.282 | 553 |  | TCA | S | 0.196 | 14.862 | 353 | trnS-UGA |
| CAC | H | 0.228 | 5.263 | 125 | trnH-GUG | TCC | S | 0.16 | 12.125 | 288 | trnS-GGA |
| CAT | H | 0.772 | 17.809 | 423 |  | TCG | S | 0.103 | 7.831 | 186 | trnS-CGA |
| ATA | I | 0.299 | 25.261 | 600 |  | TCT | S | 0.279 | 21.219 | 504 |  |
| ATC | I | 0.208 | 17.599 | 418 | trnI-GAU | ACA | T | 0.295 | 15.241 | 362 | trnT-UGU |
| ATT | I | 0.493 | 41.596 | 988 |  | ACC | T | 0.197 | 10.189 | 242 | trnT-GGU |
| AAA | K | 0.741 | 39.281 | 933 | trnK-UUU | ACG | T | 0.108 | 5.557 | 132 |  |
| AAG | K | 0.259 | 13.725 | 326 |  | ACT | T | 0.4 | 20.63 | 490 |  |
| CTA | L | 0.145 | 15.451 | 367 | trnL-UAG | GTA | V | 0.367 | 20.588 | 489 |  |
| CTC | L | 0.066 | 7.031 | 167 |  | GTC | V | 0.115 | 6.442 | 153 | trnV-GAC |
| CTG | L | 0.067 | 7.115 | 169 |  | GTG | V | 0.137 | 7.663 | 182 |  |
| CTT | L | 0.212 | 22.609 | 537 |  | GTT | V | 0.382 | 21.43 | 509 |  |
| TTA | L | 0.31 | 33.008 | 784 | trnL-UAA | TGG | W | 1 | 17.304 | 411 | trnW-CCA |
| TTG | L | 0.201 | 21.388 | 508 | trnL-CAA | TAC | Y | 0.204 | 7.284 | 173 | trnY-GUA |
| ATG | M | 1 | 23.703 | 563 | trnM-CAU | TAT | Y | 0.796 | 28.419 | 675 |  |
| AAC | N | 0.244 | 11.157 | 265 | trnN-GUU |  |  |  |  |  |  |

**Table S3. Distribution of tri-, tetra-, and penta- nucleotide SSR loci in the cp genome of *S. cusia*.**

| SSR ID | SSR Type | SSR Sequence | SSR Size | SSR Start | SSR End | SSR Location | Region |
| --- | --- | --- | --- | --- | --- | --- | --- |
| 1 | ^d^p1 | (A)8 | 8 | 1672 | 1679 | *trnK-UUU-matK* | ^a^IGS |
| 2 | p1 | (T)10 | 10 | 2952 | 2961 | *matK-trnK-UUU* | IGS |
| 3 | p1 | (A)8 | 8 | 3100 | 3107 | *matK-trnK-UUU* | IGS |
| 4 | p2 | (CT)4 | 8 | 3454 | 3461 | *matK-trnK-UUU* | IGS |
| 5 | p1 | (C)10 | 10 | 4913 | 4922 | *rps16* | ^c^Intron |
| 6 | p2 | (TA)4 | 8 | 6197 | 6204 | *rps16-trnQ-UUG* | IGS |
| 7 | p2 | (AT)4 | 8 | 6370 | 6377 | *rps16-trnQ-UUG* | IGS |
| 8 | p2 | (TA)4 | 8 | 6678 | 6685 | *trnQ-UUG-psbK* | IGS |
| 9 | p1 | (T)8 | 8 | 7075 | 7082 | *psbK* | ^b^Exon |
| 10 | p1 | (T)9 | 9 | 7500 | 7508 | *psbK-psbI* | IGS |
| 11 | p1 | (A)8 | 8 | 7711 | 7718 | *psbI-trnS-GCU* | IGS |
| 12 | ^e^c | (TA)4ttctatactatttctatc(TA)6 | 38 | 7939 | 7976 | *trnS-GCU-trnS-CGA* | IGS |
| 13 | p2 | (AT)6 | 12 | 8177 | 8188 | *trnS-GCU-trnS-CGA* | IGS |
| 14 | p5 | (ATAAA)3 | 15 | 8879 | 8893 | *trnS-CGA* | Intron |
| 15 | p1 | (T)8 | 8 | 9609 | 9616 | *trnS-CGA-trnR-UCU* | IGS |
| 16 | p1 | (A)8 | 8 | 11382 | 11389 | *atpA-atpF* | IGS |
| 17 | c | (T)10cagttcaactccaaaaaattcttcttaattatacatact(TA)4atatataaataatatataaataact(TA)4 | 90 | 12075 | 12164 | *atpF* | Intron |
| 18 | p2 | (CA)4 | 8 | 12572 | 12579 | *atpF* | Intron |
| 19 | c | (T)9gaaatcgagtcaaaaaaatattcgagttatagttataaactatgaactacatctcgattgttgccagacctcag(A)8 | 91 | 12814 | 12904 | *atpF-atpH* | IGS |
| 20 | p2 | (AT)5 | 10 | 13513 | 13522 | *atpH-atpI* | IGS |
| 21 | p4 | (ATAA)3 | 12 | 15307 | 15318 | *atpI-rps2* | IGS |
| 22 | p1 | (T)8 | 8 | 15845 | 15852 | *rps2* | Exon |
| 23 | p1 | (A)8 | 8 | 16103 | 16110 | *rps2-rpoC2* | IGS |
| 24 | p1 | (T)9 | 9 | 16220 | 16228 | *rps2-rpoC2* | IGS |
| 25 | p1 | (T)9 | 9 | 16412 | 16420 | *rpoC2* | Exon |
| 26 | p1 | (T)8 | 8 | 18303 | 18310 | *rpoC2* | Exon |
| 27 | p1 | (A)8 | 8 | 18443 | 18450 | *rpoC2* | Exon |
| 28 | p3 | (AAT)4 | 12 | 18816 | 18827 | *rpoC2* | Exon |
| 29 | p2 | (AT)5 | 10 | 19679 | 19688 | *rpoC2* | Exon |
| 30 | p1 | (A)8 | 8 | 22119 | 22126 | *rpoC1* | ^c^Exon |
| 31 | p1 | (T)9 | 9 | 22444 | 22452 | *rpoC1* | Intron |
| 32 | p1 | (T)8 | 8 | 26086 | 26093 | *rpoB* | Exon |
| 33 | p3 | (AAT)4 | 12 | 27500 | 27511 | *rpoB-trnC-GCA* | IGS |
| 34 | c | (TA)5actatatactaatatactaacactaac(TA)6 | 49 | 28149 | 28197 | *trnC-GCA-petN* | IGS |
| 35 | p1 | (T)8 | 8 | 28406 | 28413 | *trnC-GCA-petN* | IGS |
| 36 | p1 | (A)9 | 9 | 30275 | 30283 | *psbM-trnD-GUC* | IGS |
| 37 | c | (TA)4attaaatttttttatttctattataattatatattaatta(TAT)4ttaattatttataatagaaattatttatcattcatattc(AT)4aata(TAAT)3 | 123 | 31255 | 31377 | *trnE-UUC-trnT-GGU* | IGS |
| 38 | p2 | (TC)4 | 8 | 32278 | 32285 | *trnT-GGU-psbD* | IGS |
| 39 | p3 | (TTC)4 | 12 | 35175 | 35186 | *psbC* | Exon |
| 40 | p1 | (T)10 | 10 | 35427 | 35436 | *psbC-trnS-UGA* | IGS |
| 41 | p2 | (GA)4 | 8 | 35572 | 35579 | *trnS-UGA* | Exon |
| 42 | c | (C)8(A)8 | 16 | 36195 | 36210 | *psbZ-trnG-GCC* | IGS |
| 43 | p1 | (A)9 | 9 | 36325 | 36333 | *psbZ-trnG-GCC* | IGS |
| 44 | p4 | (AATT)3 | 12 | 36622 | 36633 | *trnG-GCC-trnM-CAU* | IGS |
| 45 | p1 | (T)9 | 9 | 42440 | 42448 | *psaA-ycf3* | IGS |
| 46 | c | (TA)7gaaatagaatatataagttatattaagtagaaagatactttc(TA)5 | 66 | 46336 | 46401 | *rps4-trnT-UGU* | IGS |
| 47 | c | (A)9tcgaccgttcaagtctccaaaatggaattggaaaagaagcaggaacagaaac(AT)4 | 69 | 46935 | 47003 | *trnT-UGU-trnL-UAA* | IGS |
| 48 | p1 | (A)9 | 9 | 50844 | 50852 | *ndhC-trnC-ACA* | IGS |
| 49 | p2 | (GT)4 | 8 | 51003 | 51010 | *ndhC-trnC-ACA* | IGS |
| 50 | p1 | (A)9 | 9 | 51302 | 51310 | *ndhC-trnC-ACA* | IGS |
| 51 | c | (T)10caatcgtggaaaccccagacccagaagtagtaggattaattctcataataaataatatctcataataataaaaataaat(A)9 | 98 | 54586 | 54683 | *atpB, atpB-rbcL* | Exon, IGS |
| 52 | p1 | (T)11 | 11 | 54988 | 54998 | *atpB-rbcL* | IGS |
| 53 | p2 | (GA)4 | 8 | 55878 | 55885 | *rbcL* | Exon |
| 54 | p4 | (TAGT)3 | 12 | 57202 | 57213 | *rbcL-accD* | IGS |
| 55 | p1 | (G)8 | 8 | 58658 | 58665 | *accD* | Exon |
| 56 | p2 | (TA)4 | 8 | 59341 | 59348 | *accD-psaI* | IGS |
| 57 | c | (T)8agttctacattcctttgatttcttctatatactcactcagatctaagtatc(TA)4gatacttagatctctaataagaattttcaagttgaatta(ATT)6aat(TAA)4ttaatttt(ATA)4actataaactatgaattcctaataattcacaattctgaattattattagtataattataaat(A)9 | 230 | 59561 | 59790 | *accD-psaI* | IGS |
| 58 | p1 | (T)8 | 8 | 60502 | 60509 | *ycf4* | Exon |
| 59 | p1 | (T)8 | 8 | 61325 | 61332 | *ycf4-cemA* | IGS |
| 60 | p2 | (TC)4 | 8 | 61805 | 61812 | *cemA* | Exon |
| 61 | p2 | (TA)4 | 8 | 62704 | 62711 | *petA* | Exon |
| 62 | p1 | (A)8 | 8 | 63065 | 63072 | *petA* | Exon |
| 63 | p1 | (A)8 | 8 | 63628 | 63635 | *petA-psbJ* | IGS |
| 64 | p3 | (ATA)4 | 12 | 64020 | 64031 | *petA-psbJ* | IGS |
| 65 | p1 | (A)8 | 8 | 64710 | 64717 | *psbF* | Exon |
| 66 | p4 | (TTTC)3 | 12 | 65270 | 65281 | *psbE-petL* | IGS |
| 67 | p1 | (A)9 | 9 | 67732 | 67740 | *rpl33-rps18* | IGS |
| 68 | p1 | (A)9 | 9 | 68246 | 68254 | *rps18-rpl20* | IGS |
| 69 | p2 | (CT)4 | 8 | 68474 | 68481 | *rpl20* | Exon |
| 70 | p1 | (A)9 | 9 | 68847 | 68855 | *rpl20-rps12* | IGS |
| 71 | p1 | (A)8 | 8 | 68962 | 68969 | *rpl20-rps12* | IGS |
| 72 | p1 | (T)8 | 8 | 69257 | 69264 | *rpl20-rps12* | IGS |
| 73 | p1 | (T)8 | 8 | 69404 | 69411 | *rpl20-rps12* | IGS |
| 74 | p1 | (T)8 | 8 | 69771 | 69778 | *rps12-clpP* | IGS |
| 75 | c | (T)9cgcactc(T)9 | 25 | 70331 | 70355 | *clpP* | Intron |
| 76 | p1 | (A)10 | 10 | 70485 | 70494 | *clpP* | Intron |
| 77 | p4 | (TTTC)3 | 12 | 71116 | 71127 | *clpP* | Intron |
| 78 | c | (A)8ttaaagaaaaagaaataggcaaagagaggattcaagaggcctgtaaggattaacg(A)9 | 72 | 75032 | 75103 | *petB* | Intron |
| 79 | p1 | (T)8 | 8 | 76792 | 76799 | *petD* | Intron |
| 80 | p2 | (TA)4 | 8 | 77673 | 77680 | *rpoA* | Exon |
| 81 | p1 | (A)8 | 8 | 79324 | 79331 | *rpl36-infA* | IGS |
| 82 | p1 | (T)8 | 8 | 79639 | 79646 | *infA-rps8* | IGS |
| 83 | p1 | (A)9 | 9 | 80693 | 80701 | *rpl14-rpl16* | IGS |
| 84 | c | (T)9agtgaacgtatcacagcttactcctatttttttg(T)9 | 52 | 83612 | 83663 | *rps19* | Exon |
| 85 | p2 | (TA)4 | 8 | 84244 | 84251 | *rpl2* | Intron |
| 86 | c | (GA)4tatt(GA)4tcaagaattctcactatttcttagagtcatggacccgattcaattca(T)9 | 76 | 85888 | 85963 | *trnI-CAU-ycf2* | IGS |
| 87 | p1 | (A)10 | 10 | 86118 | 86127 | *trnI-CAU-ycf2* | IGS |
| 88 | p1 | (T)10 | 10 | 86468 | 86477 | *trnI-CAU-ycf2* | IGS |
| 89 | p2 | (GA)4 | 8 | 86862 | 86869 | *ycf2* | Exon |
| 90 | c | (T)8gtgcgtcggt(A)8 | 26 | 89002 | 89027 | *ycf2* | Exon |
| 91 | p2 | (TA)4 | 8 | 92328 | 92335 | *ycf2* | Exon |
| 92 | p2 | (AG)4 | 8 | 95375 | 95382 | *ndhB* | Exon |
| 93 | p1 | (T)8 | 8 | 98002 | 98009 | *rps7-rps12* | IGS |
| 94 | p2 | (CG)4 | 8 | 98932 | 98939 | *rps12-trnV-GAC* | IGS |
| 95 | p1 | (G)8 | 8 | 102078 | 102085 | *rrn16S* | Exon |
| 96 | p1 | (T)8 | 8 | 102931 | 102938 | *trnI-GAU* | Intron |
| 97 | p1 | (G)9 | 9 | 104143 | 104151 | *trnA-UGC* | Intron |
| 98 | p2 | (CT)4 | 8 | 106271 | 106278 | *rrn23S* | Exon |
| 99 | p1 | (T)8 | 8 | 110727 | 110734 | *ndhF* | Exon |
| 100 | c | (ATGA)3attaaatttattactaatataata(AATT)3 | 48 | 112667 | 112714 | *ndhF-rpl32* | IGS |
| 101 | p5 | (AAATA)3 | 15 | 112945 | 112959 | *rpl32, rpl32-trnL-UAG* | Exon, IGS |
| 102 | c | (A)9tagtagaactatccatttttccaagaat(A)10 | 47 | 113248 | 113294 | *rpl32-trnL-UAG* | IGS |
| 103 | p1 | (A)8 | 8 | 113611 | 113618 | *trnL-UAG-ccsA* | IGS |
| 104 | p1 | (A)8 | 8 | 113914 | 113921 | *ccsA* | Exon |
| 105 | p1 | (T)8 | 8 | 114252 | 114259 | *ccsA* | Exon |
| 106 | p1 | (T)8 | 8 | 114836 | 114843 | *ccsA-ndhD* | IGS |
| 107 | p4 | (AATA)3 | 12 | 115066 | 115077 | *ndhD* | Exon |
| 108 | p1 | (T)8 | 8 | 115457 | 115464 | *ndhD* | Exon |
| 109 | p1 | (A)8 | 8 | 115953 | 115960 | *ndhD* | Exon |
| 110 | p3 | (ATT)5 | 15 | 116988 | 117002 | *psaC-ndhE* | IGS |
| 111 | p2 | (AT)4 | 8 | 119179 | 119186 | *ndhA* | Exon |
| 112 | p1 | (T)9 | 9 | 119922 | 119930 | *ndhA* | Intron |
| 113 | p4 | (CAAT)3 | 12 | 120324 | 120335 | *ndhA* | Intron |
| 114 | p1 | (A)10 | 10 | 120490 | 120499 | *ndhA* | Intron |
| 115 | p2 | (TC)4 | 8 | 122422 | 122429 | *rps15* | Exon |
| 116 | p1 | (T)8 | 8 | 123475 | 123482 | *ycf1* | Exon |
| 117 | p4 | (AATT)3 | 12 | 124694 | 124705 | *ycf1* | Exon |
| 118 | c | (TAT)4caattgacttttttctagtcgaaatcaaaaaaagaggaattgtatttttc(T)8attaagtcttctttctcttctttcattattgtaaatggatttatcaataa(T)11 | 131 | 125102 | 125232 | *ycf1* | Exon |
| 119 | c | (T)11gtc(A)9 | 23 | 125525 | 125547 | *ycf1* | Exon |
| 120 | p1 | (T)8 | 8 | 125708 | 125715 | *ycf1* | Exon |
| 121 | c | (T)10aattttctaa(T)10 | 30 | 125826 | 125855 | *ycf1* | Exon |
| 122 | p1 | (A)9 | 9 | 127476 | 127484 | *ycf1* | Exon |
| 123 | c | (T)9cctttcacttctagttcttccctttcatgtattttattcaga(TC)4 | 59 | 127747 | 127805 | *ycf1* | Exon |
| 124 | p2 | (AG)4 | 8 | 131522 | 131529 | *rrn23S* | Exon |
| 125 | p1 | (C)9 | 9 | 133649 | 133657 | *trnA-UGC* | Intron |
| 126 | p1 | (A)8 | 8 | 134862 | 134869 | *trnI-GAU* | Intron |
| 127 | p1 | (C)8 | 8 | 135715 | 135722 | *rrn16S* | Exon |
| 128 | p2 | (CG)4 | 8 | 138861 | 138868 | *trnV-GAC-rps12* | IGS |
| 129 | p1 | (A)8 | 8 | 139791 | 139798 | *rps12-rps7* | IGS |
| 130 | p2 | (CT)4 | 8 | 142418 | 142425 | *ndhB* | Exon |

^a^intergenic spacer reg**i**on; *^b^*exon sequence; ^c^intron sequence; ^d^perfect microsatellite sequences with the size of the repeat indicated after “p”; ^e^compound microsatellite sequences, which are two microsatellite sequences disrupted by a certain number of bases.

**Table S4 Distributions of tandem repeat loci in the *S. cusia* cp genome**

| ID | Indices | Size of Repeat Unit | Copy Number | Percent Matches | Location | Region | Full-length Repeat Sequence |
| --- | --- | --- | --- | --- | --- | --- | --- |
| 1 | 7919-7973 | 25 | 2.2 | 87 | *trnS-GCU-trnS-CGA* | ^a^IGS | ^d^(TATATTCTATAATTCTATACTATA)(TATATTCTATACTATTTCTATC)(TATATATAT) |
| 2 | 8476-8517 | 22 | 1.9 | 85 | *trnS-GCU-trnS-CGA* | IGS | (ATTAATATTAAAAATATTAAGA)(ATTAAGATTAAGA)(ATTTTAA) |
| 3 | 8512-8536 | 12 | 2.1 | 100 | *trnS-GCU-trnS-CGA* | IGS | (TTTTAAAGTATT)(TTTTAAAGTATT)(T) |
| 4 | 12124-12187 | 24 | 2.7 | 76 | *atpF* | ^b^Intron | (TATATATAATATATAAATAATA)(TATAAATAACTTATATATAAATA)(TATAAGATAAGGTATATAA) |
| 5 | 13466-13491 | 13 | 2 | 100 | *atpH-atpI* | IGS | (TCAAAGTAACTAA)(TCAAAGTAACTAA) |
| 6 | 22338-22378 | 20 | 2.2 | 86 | *rpoC1* | Intron | (AAATTCTGGAAACCCAAG)(AAATTATGGAAAACCCCAAG)(AAA) |
| 7 | 28133-28205 | 38 | 1.9 | 91 | *trnC-GCA-petN* | IGS | (ATATACTAATACTAACTATATATATAACTATATACTA)(ATATACTAACACTAACTATATATATATAACTATATA) |
| 8 | 31246-31322 | 36 | 2.1 | 83 | *trnE-UUC-trnT-GGU* | IGS | (ATTATATATTATATATAATTAAATTTTTTTATTTCT)(ATTATAATTATATATTAATTATATTATTATTATTTAA)(TTAT) |
| 9 | 46420-46451 | 15 | 2.1 | 94 | *rps4-trnT-UGU* | IGS | (ATAGAATATATCTAA)(ATAGAATAGATCTAA)(AT) |
| 10 | 46454-46491 | 18 | 2.1 | 90 | *rps4-trnT-UGU* | IGS | (AAAATAAGAATATAATAT)(AAAATAGAATAATAATAT)(AA) |
| 11 | 51262-51288 | 14 | 1.9 | 100 | *ndhC-trnC-ACA* | IGS | (TTAAATTCATTAAC)(TTAAATTCATTAA) |
| 12 | 54632-54663 | 16 | 1.9 | 93 | *atpB* | ^c^Exon | (TAATTCTCATAATAAA)(TAATATCTCATAATAA) |
| 13 | 60204-60247 | 21 | 2.1 | 100 | *psaI-ycf4* | IGS | (TTCACATCAAACTAGTGCTAG)(TTCACATCAAACTAG)(TGCTAGTT) |
| 14 | 66496-66520 | 12 | 2.1 | 100 | *trnW-CCA-trnP-TGG* | IGS | (AGTTTCATAAAA)(AGTTTCATAAAA)A |
| 15 | 66774-66805 | 15 | 2.1 | 100 | *trnP-TGG-psaJ* | IGS | (ATTTAACTTATAATA)(ATTTAACTTATAATA)(AT) |
| 16 | 67266-67313 | 14 | 3.3 | 88 | *psaJ-rpl33* | IGS | (AATATTAATAGAAC)(AATAATAATAAGAAC)(AATAATAATAGAAG)CAATA |
| 17 | 67824-67864 | 21 | 2 | 90 | *rps18* | Exon | (TAAATCCAAACGACCTTTTCT)(TAAATCCAAGCGATCTTTTC) |
| 18 | 88392-88457 | 24 | 2.9 | 66 | *ycf2* | Exon | (TTTGTCTAAGCCACTTCCTT)TCTTTTTGTCCAAGTTGCTGTTCTT)(TTTGTCTAGCTCACTTCCTTT) |
| 19 | 90777-90818 | 18 | 2.3 | 95 | *ycf2* | Exon | (CGATATTGATGCTAGTGG)(CGATATTGATGCTAGTGA)(CGATAT) |
| 20 | 113282-113341 | 26 | 2.4 | 77 | *rpl32-trnL-UAG* | IGS | (AATAAAAAAAAAAGGAAAGTCTAA)(AATACATAAAAAAGCGAAAGTCCTAA)(ACGAAAAAAA) |

^a^intergenic spacer reg**i**on; ^b^intronic region; *^c^*exonic region; ^d^repeat units were embraced by brackets.

**Table S5 Distribution of forward and palindromic repeat loci in the *S. cusia* cp genome.**

| Repeat ID | Repeat Type | Repeat Unit | Repeat position | Repeat Sequence | Repeat Length | Repeat Location | Repeat Region |
| --- | --- | --- | --- | --- | --- | --- | --- |
| 1 | Palindromic | Repeat | 114689-114746 | ATGAACTGGCGAGAACCCCGTGTATTCTTATAAGAATACACGGGGTTCTCGCCAGTTC  ..((((((((((((((((((((((((((....)))))))))))))))))))))))))) | 58 | *ccsA-ndhD* | IGS |
| 2 | Forward | Repeat 1 | 43639-43680 | GTTACAGAACCGTACATGAGATTTTCACCTCATACGGCTCCT | 42 | *ycf3* | intron |
|  |  | Repeat 2 | 119521-119562 | ATTACAGAACCGTACATGAGATTTTCACCTCATACGGCTCCT | 42 | *ndhA* | intron |
| 3 | Palindromic | Repeat | 46324-46359 | TTATTCTATTTCTATATATATATATAGAAATAGAAT  ..(((((((((((((((....))))))))))))))) | 36 | *rps4-trnT-UGU* | IGS |
| 4 | Palindromic | Repeat | 59606-59639 | CAGATCTAAGTATCTATATATAGATACTTAGATC  ..((((((((((((((....)))))))))))))) | 34 | *accD-psaI* | IGS |
| 5 | Forward | Repeat 1 | 43648-43680 | CCGTACATGAGATTTTCACCTCATACGGCTCCT | 33 | *ycf3* | intron |
|  |  | Repeat 2 | 98865-98897 | TCGTACATGAGATTTTCACCTCATACGGCTCCT | 33 | *rps12-trnV-GAC* | IGS |
|  |  | Repeat 3 | 119530-119562 | CCGTACATGAGATTTTCACCTCATACGGCTCCT | 33 | *ndhA* | intron |
| 6 | Palindromic | Repeat 1 | 43648-43680 | CCGTACATGAGATTTTCACCTCATACGGCTCCT  ..((((((((((((((((((((((((((((((( | 33 | *ycf3* | intron |
|  |  | Repeat 2 | 138901-138933 | CGAGGAGCCGTATGAGGTGAAAATCTCATGTAC  ..)))))))))))))))))))))))))))))))) | 33 | *trnV-GAC-rps12* | IGS |
| 7 | Palindromic | Repeat 1 | 119530-119562 | CCGTACATGAGATTTTCACCTCATACGGCTCCT  ..((((((((((((((((((((((((((((((( | 33 | *ndhA* | intron |
|  |  | Repeat 2 | 138901-138933 | CGAGGAGCCGTATGAGGTGAAAATCTCATGTAC  ..)))))))))))))))))))))))))))))))) | 33 | *trnV-GAC-rps12* | IGS |
| 8 | Palindromic repeat | Repeat 1 | 7733-7762 | AACGGAAAGAGAGGGATTCGAACCCTCGGT  ..(((((((((((((((((((((((((((( | 30 | *psbI-trnS-GCU,trnS-GCU* | IGS,CDS |
|  |  | Repeat 2 | 45152-45181 | TTACCGAGGGTTCGAATCCCTCTCTTTCCG  ..))))))))))))))))))))))))))))) | 30 | *trnS-GGA* | CDS |

**Table S6 Information of chloroplast genome used for phylogenetic analyses in this study.**

| Organism/Name | [RefSeq](http://www.ncbi.nlm.nih.gov/genome/browse/) | INSDC | Order | Size (Kb) | [GC%](http://www.ncbi.nlm.nih.gov/genome/browse/) | [Protein](http://www.ncbi.nlm.nih.gov/genome/browse/) | [rRNA](http://www.ncbi.nlm.nih.gov/genome/browse/) | [tRNA](http://www.ncbi.nlm.nih.gov/genome/browse/) | [Other RNA](http://www.ncbi.nlm.nih.gov/genome/browse/) | [Gene](http://www.ncbi.nlm.nih.gov/genome/browse/) | [Pseudogene](http://www.ncbi.nlm.nih.gov/genome/browse/) | [Release Date](http://www.ncbi.nlm.nih.gov/genome/browse/) | [Modify Date](http://www.ncbi.nlm.nih.gov/genome/browse/) |
| --- | --- | --- | --- | --- | --- | --- | --- | --- | --- | --- | --- | --- | --- |
| *Andrographis paniculata* | [NC_022451.2](http://www.ncbi.nlm.nih.gov/nuccore/NC_022451.2) | [KF150644](http://www.ncbi.nlm.nih.gov/nuccore/KF150644) | Acanthaceae | 150.249 | 38.29 | [87](http://www.ncbi.nlm.nih.gov/genome/proteins/16660?genome_assembly_id=46241&gi=685470381) | 8 | 37 | - | 132 | - | 2013/10/24 | 2014/9/17 |
| *Ruellia breedlovei* | KP300014.1 | KP300014.1 | Acanthaceae | 148.68 |  |  |  |  |  |  |  | PLN 14-MAR-2015 | |
| [*Tanaecium tetragonolobum*](http://www.ncbi.nlm.nih.gov/genome/40449?genome_assembly_id=249624) | [NC_027955.1](http://www.ncbi.nlm.nih.gov/nuccore/NC_027955.1) | [KR534325](http://www.ncbi.nlm.nih.gov/nuccore/KR534325) | Bignoniaceae | 153.776 | 38.26 | [79](http://www.ncbi.nlm.nih.gov/genome/proteins/40449?genome_assembly_id=249624&gi=937408490) | 8 | 38 | - | 126 | 1 | 2015/10/7 | 2015/10/7 |
| [*Dorcoceras hygrometricum*](http://www.ncbi.nlm.nih.gov/genome/12223?genome_assembly_id=40552) | [NC_016468.1](http://www.ncbi.nlm.nih.gov/nuccore/NC_016468.1) | [JN107811](http://www.ncbi.nlm.nih.gov/nuccore/JN107811) | Gesneriaceae | 153.493 | 37.59 | [85](http://www.ncbi.nlm.nih.gov/genome/proteins/12223?genome_assembly_id=40552&gi=364283964) | 8 | 36 | - | 145 | 16 | 2011/12/20 | 2011/12/20 |
| [*Haplostachys haplostachya HI0333*](http://www.ncbi.nlm.nih.gov/genome/44441?genome_assembly_id=273663) | [NC_029819.1](http://www.ncbi.nlm.nih.gov/nuccore/NC_029819.1) | [KU724133](http://www.ncbi.nlm.nih.gov/nuccore/KU724133) | Lamiaceae | 150.13 | 38.51 | [88](http://www.ncbi.nlm.nih.gov/genome/proteins/44441?genome_assembly_id=273663&gi=1016471368) | 16 | 74 | - | 266 | - | 2016/4/9 | 2016/4/9 |
| [*Lavandula angustifolia*](http://www.ncbi.nlm.nih.gov/genome/43769?genome_assembly_id=266370) | [NC_029370.1](http://www.ncbi.nlm.nih.gov/nuccore/NC_029370.1) | [KT948988](http://www.ncbi.nlm.nih.gov/nuccore/KT948988) | Lamiaceae | 153.448 | 38.04 | [88](http://www.ncbi.nlm.nih.gov/genome/proteins/43769?genome_assembly_id=266370&gi=1002161910) | 8 | 37 | - | 134 | 1 | 2016/3/1 | 2016/3/1 |
| [*Phyllostegia velutina HI0361*](http://www.ncbi.nlm.nih.gov/genome/44442?genome_assembly_id=273664) | [NC_029820.1](http://www.ncbi.nlm.nih.gov/nuccore/NC_029820.1) | [KU724134](http://www.ncbi.nlm.nih.gov/nuccore/KU724134) | Lamiaceae | 150.131 | 38.47 | [88](http://www.ncbi.nlm.nih.gov/genome/proteins/44442?genome_assembly_id=273664&gi=1016471457) | 16 | 74 | - | 266 | - | 2016/4/9 | 2016/4/9 |
| [*Premna microphylla*](http://www.ncbi.nlm.nih.gov/genome/36080?genome_assembly_id=218893) | [NC_026291.1](http://www.ncbi.nlm.nih.gov/nuccore/NC_026291.1) | [KM981744](http://www.ncbi.nlm.nih.gov/nuccore/KM981744) | Lamiaceae | 155.293 | 37.87 | [87](http://www.ncbi.nlm.nih.gov/genome/proteins/36080?genome_assembly_id=218893&gi=752789765) | 8 | 37 | - | 133 | - | 2015/2/4 | 2015/2/4 |
| [*Rosmarinus officinalis*](http://www.ncbi.nlm.nih.gov/genome/12899?genome_assembly_id=232708) | [NC_027259.1](http://www.ncbi.nlm.nih.gov/nuccore/NC_027259.1) | [KR232566](http://www.ncbi.nlm.nih.gov/nuccore/KR232566) | Lamiaceae | 152.462 | 37.99 | [86](http://www.ncbi.nlm.nih.gov/genome/proteins/12899?genome_assembly_id=232708&gi=836643369) | 8 | 37 | - | 134 | 3 | 2015/6/18 | 2015/6/18 |
| [*Salvia miltiorrhiza*](http://www.ncbi.nlm.nih.gov/genome/11235?genome_assembly_id=40000) | [NC_020431.1](http://www.ncbi.nlm.nih.gov/nuccore/NC_020431.1) | [JX312195](http://www.ncbi.nlm.nih.gov/nuccore/JX312195) | Lamiaceae | 151.328 | 38.02 | [87](http://www.ncbi.nlm.nih.gov/genome/proteins/11235?genome_assembly_id=40000&gi=459014471) | 8 | 37 | - | 133 | 1 | 2013/3/4 | 2013/3/4 |
| [*Scutellaria baicalensis*](http://www.ncbi.nlm.nih.gov/genome/38543?genome_assembly_id=232783) | [NC_027262.1](http://www.ncbi.nlm.nih.gov/nuccore/NC_027262.1) | [KR233163](http://www.ncbi.nlm.nih.gov/nuccore/KR233163) | Lamiaceae | 152.731 | 38.38 | [87](http://www.ncbi.nlm.nih.gov/genome/proteins/38543?genome_assembly_id=232783&gi=836643484) | 8 | 36 | - | 132 | 1 | 2015/6/18 | 2015/8/4 |
| [*Scutellaria insignis*](http://www.ncbi.nlm.nih.gov/genome/41855?genome_assembly_id=259429) | [NC_028533.1](http://www.ncbi.nlm.nih.gov/nuccore/NC_028533.1) | [KT750009](http://www.ncbi.nlm.nih.gov/nuccore/KT750009) | Lamiaceae | 151.908 | 38.38 | [87](http://www.ncbi.nlm.nih.gov/genome/proteins/41855?genome_assembly_id=259429&gi=959123030) | 8 | 37 | - | 133 | 1 | 2015/12/3 | 2015/12/3 |
| [*Stachys byzantina CL1004*](http://www.ncbi.nlm.nih.gov/genome/44465?genome_assembly_id=273687) | [NC_029825.1](http://www.ncbi.nlm.nih.gov/nuccore/NC_029825.1) | [KU724141](http://www.ncbi.nlm.nih.gov/nuccore/KU724141) | Lamiaceae | 149.749 | 38.7 | [88](http://www.ncbi.nlm.nih.gov/genome/proteins/44465?genome_assembly_id=273687&gi=1016471902) | 16 | 74 | - | 266 | - | 2016/4/9 | 2016/4/9 |
| [*Stachys chamissonis CL1002*](http://www.ncbi.nlm.nih.gov/genome/44468?genome_assembly_id=273690) | [NC_029822.1](http://www.ncbi.nlm.nih.gov/nuccore/NC_029822.1) | [KU724138](http://www.ncbi.nlm.nih.gov/nuccore/KU724138) | Lamiaceae | 150.254 | 38.53 | [88](http://www.ncbi.nlm.nih.gov/genome/proteins/44468?genome_assembly_id=273690&gi=1016471635) | 16 | 74 | - | 266 | - | 2016/4/9 | 2016/4/9 |
| [*Stachys coccinea CL1003*](http://www.ncbi.nlm.nih.gov/genome/44443?genome_assembly_id=273665) | [NC_029823.1](http://www.ncbi.nlm.nih.gov/nuccore/NC_029823.1) | [KU724139](http://www.ncbi.nlm.nih.gov/nuccore/KU724139) | Lamiaceae | 150.275 | 38.5 | [88](http://www.ncbi.nlm.nih.gov/genome/proteins/44443?genome_assembly_id=273665&gi=1016471724) | 16 | 74 | - | 266 | - | 2016/4/9 | 2016/4/9 |
| [*Stachys sylvatica LVA358*](http://www.ncbi.nlm.nih.gov/genome/44450?genome_assembly_id=273672) | [NC_029824.1](http://www.ncbi.nlm.nih.gov/nuccore/NC_029824.1) | [KU724140](http://www.ncbi.nlm.nih.gov/nuccore/KU724140) | Lamiaceae | 150.167 | 38.56 | [88](http://www.ncbi.nlm.nih.gov/genome/proteins/44450?genome_assembly_id=273672&gi=1016471813) | 16 | 74 | - | 266 | - | 2016/4/9 | 2016/4/9 |
| [*Stenogyne bifida HI0332*](http://www.ncbi.nlm.nih.gov/genome/44444?genome_assembly_id=273666) | [NC_029818.1](http://www.ncbi.nlm.nih.gov/nuccore/NC_029818.1) | [KU724132](http://www.ncbi.nlm.nih.gov/nuccore/KU724132) | Lamiaceae | 150.129 | 38.51 | [88](http://www.ncbi.nlm.nih.gov/genome/proteins/44444?genome_assembly_id=273666&gi=1016471279) | 16 | 74 | - | 266 | - | 2016/4/9 | 2016/4/9 |
| [*Stenogyne haliakalae GEO5*](http://www.ncbi.nlm.nih.gov/genome/44445?genome_assembly_id=273667) | [NC_029817.1](http://www.ncbi.nlm.nih.gov/nuccore/NC_029817.1) | [KU724130](http://www.ncbi.nlm.nih.gov/nuccore/KU724130) | Lamiaceae | 149.736 | 38.53 | [88](http://www.ncbi.nlm.nih.gov/genome/proteins/44445?genome_assembly_id=273667&gi=1016471190) | 16 | 74 | - | 266 | - | 2016/4/9 | 2016/4/9 |
| [*Stenogyne kanehoana HSJ14046*](http://www.ncbi.nlm.nih.gov/genome/44446?genome_assembly_id=273668) | [NC_029821.1](http://www.ncbi.nlm.nih.gov/nuccore/NC_029821.1) | [KU724136](http://www.ncbi.nlm.nih.gov/nuccore/KU724136) | Lamiaceae | 150.115 | 38.53 | [88](http://www.ncbi.nlm.nih.gov/genome/proteins/44446?genome_assembly_id=273668&gi=1016471546) | 16 | 74 | - | 266 | - | 2016/4/9 | 2016/4/9 |
| [*Tectona grandis*](http://www.ncbi.nlm.nih.gov/genome/16024?genome_assembly_id=248253) | [NC_020098.1](http://www.ncbi.nlm.nih.gov/nuccore/NC_020098.1) | [HF567869](http://www.ncbi.nlm.nih.gov/nuccore/HF567869) | Lamiaceae | 153.953 | 37.89 | [86](http://www.ncbi.nlm.nih.gov/genome/proteins/16024?genome_assembly_id=248253&gi=442742942) | 8 | 37 | - | 134 | 3 | 2013/1/2 | 2013/1/22 |
| [*Genlisea margaretae*](http://www.ncbi.nlm.nih.gov/genome/34686?genome_assembly_id=213301) | [NC_025652.1](http://www.ncbi.nlm.nih.gov/nuccore/NC_025652.1) | [HG530134](http://www.ncbi.nlm.nih.gov/nuccore/HG530134) | Lentibulariaceae | 141.255 | 38.34 | [56](http://www.ncbi.nlm.nih.gov/genome/proteins/34686?genome_assembly_id=213301&gi=723456729) | 8 | 36 | - | 106 | 3 | 2014/11/3 | 2014/11/18 |
| [*Pinguicula ehlersiae*](http://www.ncbi.nlm.nih.gov/genome/24248?genome_assembly_id=53443) | [NC_023463.1](http://www.ncbi.nlm.nih.gov/nuccore/NC_023463.1) | [HG803178](http://www.ncbi.nlm.nih.gov/nuccore/HG803178) | Lentibulariaceae | 147.147 | 38.19 | [72](http://www.ncbi.nlm.nih.gov/genome/proteins/24248?genome_assembly_id=53443&gi=587005057) | 8 | 37 | - | 127 | - | 2014/1/23 | 2014/2/24 |
| [*Utricularia gibba*](http://www.ncbi.nlm.nih.gov/genome/16713?genome_assembly_id=46255) | [NC_021449.1](http://www.ncbi.nlm.nih.gov/nuccore/NC_021449.1) | [KC997777](http://www.ncbi.nlm.nih.gov/nuccore/KC997777) | Lentibulariaceae | 152.113 | 37.57 | [87](http://www.ncbi.nlm.nih.gov/genome/proteins/16713?genome_assembly_id=46255&gi=519704483) | 8 | 37 | - | 133 | 1 | 2013/7/2 | 2013/7/2 |
| *Utricularia macrorhiza* | [NC_025653.1](http://www.ncbi.nlm.nih.gov/nuccore/NC_025653.1) | [HG803177](http://www.ncbi.nlm.nih.gov/nuccore/HG803177) | Lentibulariaceae | 153.228 | 37.04 | [77](http://www.ncbi.nlm.nih.gov/genome/proteins/35309?genome_assembly_id=214062&gi=728792356) | 8 | 37 | - | 135 | - | 2014/11/3 | 2014/12/3 |
| *Utricularia reniformis* | [NC_029719.1](http://www.ncbi.nlm.nih.gov/nuccore/NC_029719.1) | - | Lentibulariaceae | 148.95 | 37.76 | [75](http://www.ncbi.nlm.nih.gov/genome/proteins/44110?genome_assembly_id=271930&gi=1009104607) | 8 | 31 | - | 140 | 26 | 2016/3/24 | 2016/3/25 |
| [*Hesperelaea palmeri*](http://www.ncbi.nlm.nih.gov/genome/35376?genome_assembly_id=214262) | [NC_025787.1](http://www.ncbi.nlm.nih.gov/nuccore/NC_025787.1) | [LN515489](http://www.ncbi.nlm.nih.gov/nuccore/LN515489) | Oleaceae | 155.82 | 37.81 | [85](http://www.ncbi.nlm.nih.gov/genome/proteins/35376?genome_assembly_id=214262&gi=731971810) | 8 | 37 | - | 130 | - | 2014/11/24 | 2014/12/11 |
| [*Jasminum nudiflorum*](http://www.ncbi.nlm.nih.gov/genome/8034?genome_assembly_id=37840) | [NC_008407.1](http://www.ncbi.nlm.nih.gov/nuccore/NC_008407.1) | [DQ673255](http://www.ncbi.nlm.nih.gov/nuccore/DQ673255) | Oleaceae | 165.121 | 37.98 | [85](http://www.ncbi.nlm.nih.gov/genome/proteins/8034?genome_assembly_id=37840&gi=115391881) | 8 | 38 | - | 131 | - | 2006/9/29 | 2009/12/4 |
| [*Olea europaea*](http://www.ncbi.nlm.nih.gov/genome/10724?genome_assembly_id=39627) | [NC_013707.2](http://www.ncbi.nlm.nih.gov/nuccore/NC_013707.2) | [GU228899](http://www.ncbi.nlm.nih.gov/nuccore/GU228899) | Oleaceae | 155.888 | 37.8 | [85](http://www.ncbi.nlm.nih.gov/genome/proteins/10724?genome_assembly_id=39627&gi=365823312) | 8 | 37 | - | 130 | - | 2010/1/12 | 2011/12/28 |
| [*Olea europaea subsp. cuspidata*](http://www.ncbi.nlm.nih.gov/genome/10724?genome_assembly_id=39626) | [NC_015604.1](http://www.ncbi.nlm.nih.gov/nuccore/NC_015604.1) | [FN650747](http://www.ncbi.nlm.nih.gov/nuccore/FN650747) | Oleaceae | 155.862 | 37.81 | [85](http://www.ncbi.nlm.nih.gov/genome/proteins/10724?genome_assembly_id=39626&gi=334700261) | 8 | 37 | - | 130 | - | 2011/5/25 | 2011/8/1 |
| [*Olea europaea subsp. maroccana*](http://www.ncbi.nlm.nih.gov/genome/10724?genome_assembly_id=39624) | [NC_015623.1](http://www.ncbi.nlm.nih.gov/nuccore/NC_015623.1) | [FN998900](http://www.ncbi.nlm.nih.gov/nuccore/FN998900) | Oleaceae | 155.896 | 37.81 | [85](http://www.ncbi.nlm.nih.gov/genome/proteins/10724?genome_assembly_id=39624&gi=334701867) | 8 | 37 | - | 130 | - | 2011/5/25 | 2011/8/1 |
| [*Olea woodiana subsp. woodiana*](http://www.ncbi.nlm.nih.gov/genome/9881?genome_assembly_id=39199) | [NC_015608.1](http://www.ncbi.nlm.nih.gov/nuccore/NC_015608.1) | [FN998901](http://www.ncbi.nlm.nih.gov/nuccore/FN998901) | Oleaceae | 155.942 | 37.79 | [85](http://www.ncbi.nlm.nih.gov/genome/proteins/9881?genome_assembly_id=39199&gi=334701598) | 8 | 37 | - | 130 | - | 2011/5/25 | 2011/8/1 |
| *Cistanche deserticola* | [NC_021111.1](http://www.ncbi.nlm.nih.gov/nuccore/NC_021111.1) | [KC128846](http://www.ncbi.nlm.nih.gov/nuccore/KC128846) | Orobanchaceae | 102.657 | 36.78 | [31](http://www.ncbi.nlm.nih.gov/genome/proteins/17587?genome_assembly_id=46798&gi=501595124) | 8 | 36 | - | 106 | 31 | 2013/5/17 | 2013/5/17 |
| [*Epifagus virginiana*](http://www.ncbi.nlm.nih.gov/genome/7369?genome_assembly_id=37478) | [NC_001568.1](http://www.ncbi.nlm.nih.gov/nuccore/NC_001568.1) | [M81884](http://www.ncbi.nlm.nih.gov/nuccore/M81884) | Orobanchaceae | 70.028 | 36 | [25](http://www.ncbi.nlm.nih.gov/genome/proteins/7369?genome_assembly_id=37478&gi=11466954) | 8 | 23 | - | 71 | 15 | 1993/8/3 | 2009/5/6 |
| [*Lathraea squamaria*](http://www.ncbi.nlm.nih.gov/genome/40145?genome_assembly_id=248171) | [NC_027838.1](http://www.ncbi.nlm.nih.gov/nuccore/NC_027838.1) | [KM652488](http://www.ncbi.nlm.nih.gov/nuccore/KM652488) | Orobanchaceae | 150.504 | 38.13 | [49](http://www.ncbi.nlm.nih.gov/genome/proteins/40145?genome_assembly_id=248171&gi=927372274) | 8 | 37 | - | 130 | 36 | 2015/9/14 | 2015/9/14 |
| [*Lindenbergia philippensis*](http://www.ncbi.nlm.nih.gov/genome/23205?genome_assembly_id=48446) | [NC_022859.1](http://www.ncbi.nlm.nih.gov/nuccore/NC_022859.1) | [HG530133](http://www.ncbi.nlm.nih.gov/nuccore/HG530133) | Orobanchaceae | 155.103 | 37.79 | [85](http://www.ncbi.nlm.nih.gov/genome/proteins/23205?genome_assembly_id=48446&gi=558603845) | 8 | 37 | - | 137 | - | 2013/11/5 | 2013/11/19 |
| [*Orobanche californica*](http://www.ncbi.nlm.nih.gov/genome/34683?genome_assembly_id=213298) | [NC_025651.1](http://www.ncbi.nlm.nih.gov/nuccore/NC_025651.1) | [HG515539](http://www.ncbi.nlm.nih.gov/nuccore/HG515539) | Orobanchaceae | 120.84 | 36.69 | [45](http://www.ncbi.nlm.nih.gov/genome/proteins/34683?genome_assembly_id=213298&gi=723457210) | 8 | 41 | - | 123 | 29 | 2014/11/3 | 2014/11/18 |
| [*Orobanche crenata*](http://www.ncbi.nlm.nih.gov/genome/34174?genome_assembly_id=211380) | [NC_024845.1](http://www.ncbi.nlm.nih.gov/nuccore/NC_024845.1) | [HG515537](http://www.ncbi.nlm.nih.gov/nuccore/HG515537) | Orobanchaceae | 87.529 | 35.19 | [36](http://www.ncbi.nlm.nih.gov/genome/proteins/34174?genome_assembly_id=211380&gi=695101650) | 8 | 39 | - | 106 | 22 | 2014/9/2 | 2014/10/7 |
| *Orobanche gracilis* | [NC_023464.1](http://www.ncbi.nlm.nih.gov/nuccore/NC_023464.1) | [HG803179](http://www.ncbi.nlm.nih.gov/nuccore/HG803179) | Orobanchaceae | 65.533 | 34.56 | [24](http://www.ncbi.nlm.nih.gov/genome/proteins/24247?genome_assembly_id=53442&gi=587005157) | 7 | 31 | - | 77 | - | 2014/1/23 | 2014/2/24 |
| *Pedicularis ishidoyana* | [NC_029700.1](http://www.ncbi.nlm.nih.gov/nuccore/NC_029700.1) | [KU170194](http://www.ncbi.nlm.nih.gov/nuccore/KU170194) | Orobanchaceae | 152.571 | 38.09 | [77](http://www.ncbi.nlm.nih.gov/genome/proteins/44127?genome_assembly_id=272027&gi=1011056414) | 8 | 38 | - | 123 | - | 2016/3/28 | 2016/3/28 |
| [*Schwalbea americana*](http://www.ncbi.nlm.nih.gov/genome/24093?genome_assembly_id=50220) | [NC_023115.1](http://www.ncbi.nlm.nih.gov/nuccore/NC_023115.1) | [HG738866](http://www.ncbi.nlm.nih.gov/nuccore/HG738866) | Orobanchaceae | 160.91 | 38.08 | [82](http://www.ncbi.nlm.nih.gov/genome/proteins/24093?genome_assembly_id=50220&gi=575669189) | 8 | 37 | - | 129 | 2 | 2013/11/26 | 2014/1/28 |
| *Phelipanche purpurea* | [NC_023132.1](http://www.ncbi.nlm.nih.gov/nuccore/NC_023132.1) | [HG515536](http://www.ncbi.nlm.nih.gov/nuccore/HG515536) | Orobanchaceae; | 62.891 | 31.08 | [30](http://www.ncbi.nlm.nih.gov/genome/proteins/23971?genome_assembly_id=49398&gi=568246815) | 4 | 26 | - | 76 | 14 | 2013/12/16 | 2013/12/20 |
| [*Phelipanche ramosa*](http://www.ncbi.nlm.nih.gov/genome/24237?genome_assembly_id=53439) | [NC_023465.1](http://www.ncbi.nlm.nih.gov/nuccore/NC_023465.1) | [HG803180](http://www.ncbi.nlm.nih.gov/nuccore/HG803180) | Orobanchaceae; | 62.304 | 32.06 | [27](http://www.ncbi.nlm.nih.gov/genome/proteins/24237?genome_assembly_id=53439&gi=586695135) | 5 | 24 | - | 67 | - | 2014/1/23 | 2014/2/20 |
| [*Sesamum indicum*](http://www.ncbi.nlm.nih.gov/genome/11560?genome_assembly_id=49670) | [NC_016433.2](http://www.ncbi.nlm.nih.gov/nuccore/NC_016433.2) | - | Pedaliaceae | 153.324 | 38.2 | [87](http://www.ncbi.nlm.nih.gov/genome/proteins/11560?genome_assembly_id=49670&gi=378758475) | 8 | 37 | - | 134 | 2 | 2011/12/6 | 2015/8/4 |
| *Scrophularia takesimensis* | NC_026202.1 | [KM590983](https://www.ncbi.nlm.nih.gov/nuccore/KM590983) | Scrophulariaceae; | 152.42 | 38.05 | [88](https://www.ncbi.nlm.nih.gov/genome/proteins/36079?genome_assembly_id=218892&gi=752789663) | 8 | 36 | - | 132 | - | 2015/2/4 | 2015/2/10 |
